# Resolving the polycistronic aftermath: Essential role of topoisomerase IA in preventing R-loops in *Leishmania*

**DOI:** 10.1016/j.jbc.2024.107162

**Published:** 2024-03-12

**Authors:** Payel Das, Arnab Hazra, Saradindu Saha, Sadhana Roy, Mandrita Mukherjee, Saugata Hazra, Hemanta K. Majumdar, Somdeb BoseDasgupta

**Affiliations:** 1 Molecular Immunology and Cellular Microbiology Laboratory, Department of Bioscience and Biotechnology, Indian Institute of Technology Kharagpur, Kharagpur, India; 2 Department of Biosciences and Bioengineering, Indian Institute of Technology Roorkee, Roorkee, India; 3 Infectious Diseases and Immunology Division, CSIR- Indian Institute of Chemical Biology, Kolkata, India

**Keywords:** topoisomerase IA, polycistronic, R-loop, norclomipramine, nuclear

## Abstract

Kinetoplastid parasites are “living bridges” in the evolution from prokaryotes to higher eukaryotes. The near-intronless genome of the kinetoplastid *Leishmania* exhibits polycistronic transcription which can facilitate R-loop formation. Therefore, to prevent such DNA-RNA hybrids, *Leishmania* has retained prokaryotic-like DNA Topoisomerase IA (LdTOPIA) in the course of evolution. LdTOPIA is an essential enzyme that is expressed ubiquitously and is adapted for the compartmentalized eukaryotic form in harboring functional bipartite nuclear localization signals. Although exhibiting greater homology to mycobacterial TOPIA, LdTOPIA could functionally complement the growth lethality of *Escherichia coli* TOPIA null GyrB ts strain at non-permissive temperatures. Purified LdTOPIA exhibits Mg^2+^-dependent relaxation of only negatively supercoiled DNA and preference towards single-stranded DNA substrates. LdTOPIA prevents nuclear R-loops as conditional LdTOPIA downregulated parasites exhibit R-loop formation and thereby parasite killing. The clinically used tricyclic antidepressant, norclomipramine could specifically inhibit LdTOPIA and lead to R-loop formation and parasite elimination. This comprehensive study therefore paves an avenue for drug repurposing against *Leishmania*.

Evolutionarily kinetoplastids are a living bridge between the prokaryotes and higher eukaryotes. It has adapted to the compartmentalized structure of eukaryotes, yet harbors several prokaryote homologous enzymes and processes. Owing to a near intron-less genome kinetoplastids especially old-world species, *Leishmania* initially exhibits polycistronic transcription similar to prokaryotes but later these transcripts, exhibit trans-splicing using the spliced leader RNA to generate gene-specific mature RNA transcripts (1, 2). Although *Leishmania* and *Trypanosoma* come under kinetoplastids there are certain genome composition, functionality, and transcriptional differences between the two organisms. Trypanosomes harbor a lot more intergenic strand switch regions as compared to *Leishmania*, and this also allows for shorter nuclear transcripts compared to *Leishmania* (1, 2). The large number of long terminal repeats (LTR) and non-LTR retroelements present in the *Trypanosoma* genome as compared to *Leishmania* genome enables it to produce shorter transcripts from its tandemly arranged intron-less genes (2). Besides, the *Trypanosoma* genome harbors piwi and argonaut genes required for RNA interference while the same is absent in the old-world *Leishmania* species (1, 3).

Topoisomerases solve all the topological problems that are related to the physical structure of the DNA double helix by cleaving, manipulating, and religating DNA strands in the same catalytic event. There are two types of Topoisomerases, type I, which cleaves single-stranded DNA, and type II, which cleaves double-stranded DNA, and requires ATP for their activity. Based on the sequence and catalytic mechanism, Type I topoisomerases are divided into three subfamilies: type IA, type IB, and type IC (4, 5). Type IA includes topoisomerase IA and topoisomerase III where the enzymes require Mg^2+^ for their activity and form 5′-phosphotyrosine catalytic intermediate. *Escherichia coli* topoisomerase IA is an ideal example of a type IA enzyme involved in DNA relaxation activities and which comprises a core domain harboring the active site residues, and a C-terminal domain harboring zinc-finger motifs (4). On the other hand, Topoisomerase III is more efficient in DNA decatenation, unknotting, and resolution of recombination intermediates (4). While evolutionarily TOPIII evolved from prokaryotes to eukaryotes, TOPIA was gradually lost in evolution, hence it is absent in higher eukaryotes but can be found in lower eukaryotes like kinetoplastids (6).

*E. coli* has a global supercoiling-based regulation system mainly based on the antagonistic actions of topoisomerase I and DNA gyrase where the former relaxes and the latter generates negative supercoils (7). In normal conditions, transcription elongation generates negative and positive supercoils upstream and downstream of the moving transcription bubble harboring the RNA polymerase complex. But in extreme cases, as in the absence of topoisomerase I, transcription elongation generates hypernegatively supercoiled DNA ahead of the transcription bubble. This is generated by DNA gyrase ahead of the transcription bubble, where it removes the positive supercoils and thereafter starts to generate negative supercoils (8, 9). D-loops and R-loops are bubble-like structures that form when one strand of the double helix is displaced by annealing of the complementary strand of either DNA (D-loop) or RNA (R-loop) with the newly synthesized DNA or RNA strand (10, 11). In prokaryotes recombination events as well as plasmid replication go through an intermediate D-loop formation whereas polycistronic transcription induces, hypernegative supercoiling and results in the hybridization of nascent RNA with template DNA to generate R-loops. An R-loop is a three-stranded nucleic acid structure that consists of a stretch of DNA: RNA hybrid formed between the nascent transcribed RNA and the coding DNA strand while the non-coding strand remains displaced in the transcription bubble. R-loops are predominantly formed due to hypernegative supercoiling induced by Type II topoisomerases ahead of the transcription bubble in prokaryotes (7, 11) and they are known to act as transcriptional brakes thus generating DNA breaks, which then lead to genome instability (12, 13). The presence of efficient relaxes like topoisomerase IA in prokaryotes prevents the formation of R-loops (8). Besides, RNaseH overexpression can also suppress R-loop formation (14). But how do eukaryotes deal with this problem? For higher eukaryotes, recombination events are strictly monitored by topoisomerase IIIα and IIIβ and transcription is monocistronic thus minimizing the problems associated with hypernegative supercoiling (15, 16). But what happens for the intron-less kinetoplastids where transcription is polycistronic. Trypanosomes by virtue of having intergenic strand-switch regions, LTR and non-LTR sequences somewhat produce shorter transcripts. But how *Leishmania* deals with the problem associated with hypernegative supercoiling owing to its long polycistronic transcripts is unknown. The absence of introns, polycistronic transcription, and uncoupled transcription and translation in kinetoplastids as compared to prokaryotes are easy sources for generating R-loops, which would be fatal for the parasites (1, 17, 18, 19).

We find that *Leishmania* harbors a prokaryotic type 1A DNA Topoisomerase (LdTOPIA) homolog which is an essential enzyme capable of preventing nuclear R-loops during transcription and exhibiting Mg^2+^-dependent relaxation of only negatively supercoiled DNA. Parasites are symbolized for their adaptability and so *Leishmania* has adapted the DNA Topoisomerase IA to localize in the nucleus so as to resolve the topological constraints arising out of polycistronic transcription-associated hypernegative supercoiling and thereby prevent R-loop formation. Additionally, we have identified FDA-approved tricyclic anti-depressant norclomipramine to be a potent inhibitor of LdTOPIA and an efficient anti-leishmanial drug that can specifically be used for drug repurposing.

## Results

### DNA topoisomerase IA of *Leishmania* is a ubiquitously expressed essential enzyme

**Figure 1:**
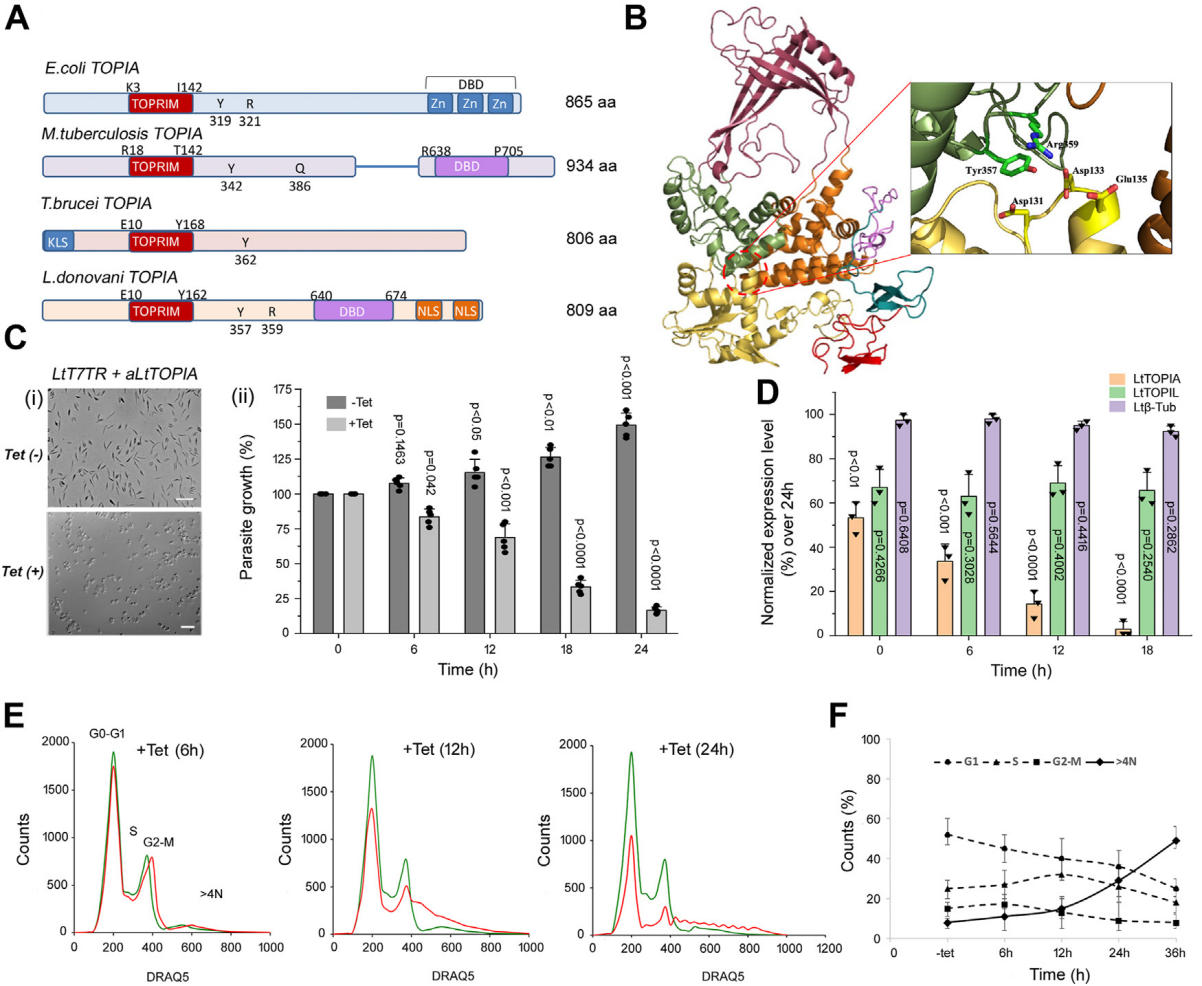
**Topoisomerase IA, an essential protein of *L. donovani*.***A*, sequence comparison of *E. coli* TOPIA, *M. tuberculosis* TOPIA, *T. brucei* TOPIA and *L. donovani* TOPIA showing start and end residue of TOPRIM domain, Active site tyrosine and DNA binding domain region. *B*, homology modeled structure of LdTOPIA with the active site residues Tyr357, Glu135, Asp131, and Asp 133 exhibited in the zoomed image. *C*, microscopic images of (−Tet) tetracycline uninduced (top) and (+Tet) induced (*bottom*) LtT7TR parasites expressing antisense LdTOPIA construct, Scale bar: 25 μm (ii) Graphical representation of percentage viable LtT7TR parasites in (−Tet) and (+Tet) condition for indicated time points. (n = 5 mean ± SD, 3 biological replicates. p vs. respective control (0h)). *D*, relative quantitation of LtTOPIA, LtTOPIL, and Ltβ-Tub mRNA expression levels in (+Tet) parasites measured by qPCR and plotted as normalized values over 24 h (n = 3 mean ± SD, 3 biological replicates. p *versus* tetracycline treated for 24 h). *E*, flow cytometric analysis of cell-cycle arrest in antisense LtTOPIA transfected LtT7TR parasites without (−Tet, *green*) or with (+Tet, *red*) induction at indicated timepoints (representative image of n = 3). *F*, graphical representation of the cell cycle phases (G_0_-G_1_, S, G_2-_M, and 4N) for antisense LtTOPIA transfected LtT7TR parasites without (−Tet) or with (+Tet) induction for indicated time points (n = 5, mean ± SD, 3 biological replicates for each time).

Vital cellular processes like replication and transcription encounter topological entanglements, which then are resolved by topoisomerases. Interestingly, the *Leishmania donovani* genome harbors a novel “prokaryotic TOPIA” homolog LdBPK_210180.1, which is absent in the higher eukaryotes. Bioinformatic analysis of *Leishmania donovani* TOPIA (LdTOPIA) shows that it harbors an active motif “GYITYPRTDST” similar to *E. coli* Topoisomerase I (EcTOPIA), but instead of having a zinc finger domain in its C-terminus, there are bipartite nuclear localization signals (Fig. 1*A*). Gene expression analysis of LdTOPIA using RNA isolated from both promastigotes and axenic amastigotes at the indicated time points exhibited equal levels at both stages of their life cycle (Fig. S1*A*). RNA isolated during the conversion of promastigotes to axenic amastigotes or vice versa did not exhibit any significant change in the expression of LdTOPIA, thus indicating that it is expressed ubiquitously at all life cycle stages of *Leishmania*. Phylogenetic analysis of topoisomerases from different species belonging to the ‘Type 1A’ class of topoisomerases exhibits that LdTOPIA belongs to the TOPIA class of topoisomerases that are found in prokaryotes, lower eukaryotes, and plants (Fig. S1*B*). Hence, we next generated a homology modeled structure of LdTOPIA using the available closely related mycobacterial TOPIA (MtTOPIA) structure (PDB ID: 5D5H). It was observed that the LdTOPIA structure resembled closely to the mycobacterial structure (Fig. 1*B*), and active site residues, highlighted in the structure are Tyr357, Glu135, Asp131, and Asp133. Superposition of the homology-modeled structure over the available *E. coli* TOPIA structure (PDB id: 4RUL) exhibited close overlap except for the zinc-finger domains that were present in *E. coli* TOPIA (Fig. S1*C*).

To observe any phenotypic and/or metabolic changes associated with LdTOPIA, we next wanted to generate LdTOPIA knockout parasites through homologous recombination. First, we replaced one LdTOPIA allele with hygromycin (Fig. S1*D*-i). Not only was the growth of these promastigotes slowed but they appeared slightly bulged and oval in shape as observed when stressed (Fig. S1*D-*ii). Next, when these heterozygous promastigotes were transfected with another allele replacing construct-containing GFP, the transfectant promastigotes failed to survive even on repeated trials (Fig. S1*D-*ii). From this, we deciphered that LdTOPIA could be an essential gene and therefore we were unable to generate a complete knockout. Since RNA interference is a set-back for the old world *Leishmania* species like *Leishmania* *donovani*, *Leishmania major,* and *Leishmania tarentolae*, and our knockdown of LdTOPIA was not possible owing to it being an essential enzyme. The only option we had was to carry out conditional antisense. The conditional antisense strategy uses a commercially available *L. tarentolae* (LtT7TR) strain (Jena Bioscience) wherein the tetracycline repressor (TR) and the T7 RNA polymerase (T7) is integrated into the genome of this strain under the control of hygromycin and nourseothricin selectable markers, respectively (Fig. S1*E*-i) (20, 21). When an antisense construct directed against the untranslated regions of LtTOPIA is cloned in a T7 promoter and tet operator containing vector such as pLew100v5 (Fig. S1*E*-ii) (22, 23), and there upon when this recombinant plasmid is transfected into this LtT7TR strain it would facilitate tetracycline induced conditional antisense of LtTOPIA and in parallel allow for complementation using either LdTOPIA or similar constructs cloned and transfected in another vector. Unfortunately, since no LdT7TR strain was available commercially and multiple sequence analyses of LtTOPIA and LdTOPIA exhibited 87.8% identity and 95.8% similarity at the sequence level, we decided that the antisense of LtTOPIA unequivocally would represent the antisense of LdTOPIA wherein complementation could be carried out with LdTOPIA.

The tetracycline-treated antiTOPIA expressing parasites exhibited round stressed morphology (Fig. 1*C-*i). A modified MTT assay using tet untreated and treated cells exhibited a gradual decrease in the viability of the parasites upon tetracycline treatment (Fig. 1*C-*ii) compared to untreated parasites due to a gradual decrease in the level of LtTOPIA transcripts with increasing time of tetracycline treatment. Analysis of the LtTOPIA transcripts at different timepoints after tetracycline treatment normalized against the expression at 24 h exhibited a gradual decrease in the level of LtTOPIA transcript as compared to LtTOPIL and β-tubulin controls. It was observed that LtTOPIA transcripts were reduced to ∼10 to 12 fold in the tetracycline-treated antisense TOPIA LtT7TR transfectant parasites compared to tetracycline untreated parasites after 24 h of treatment (Fig. 1*D*). To analyze the effect of LtTOPIA depletion on cell cycle progression flow cytometry analysis was carried out (Fig. 1*E*). Reduction of parasites, both with 2C content (G1 phase) and 4C content (G2/M phase) decreased with time of tetracycline treatment, but the percentage of parasites with more than 4C increases with time which is indicative of G2/M arrest (Fig. 1*F*), failed mitosis followed by parasite death.

### *Leishmania* topoisomerase IA localizes in the nucleus

**Figure 2:**
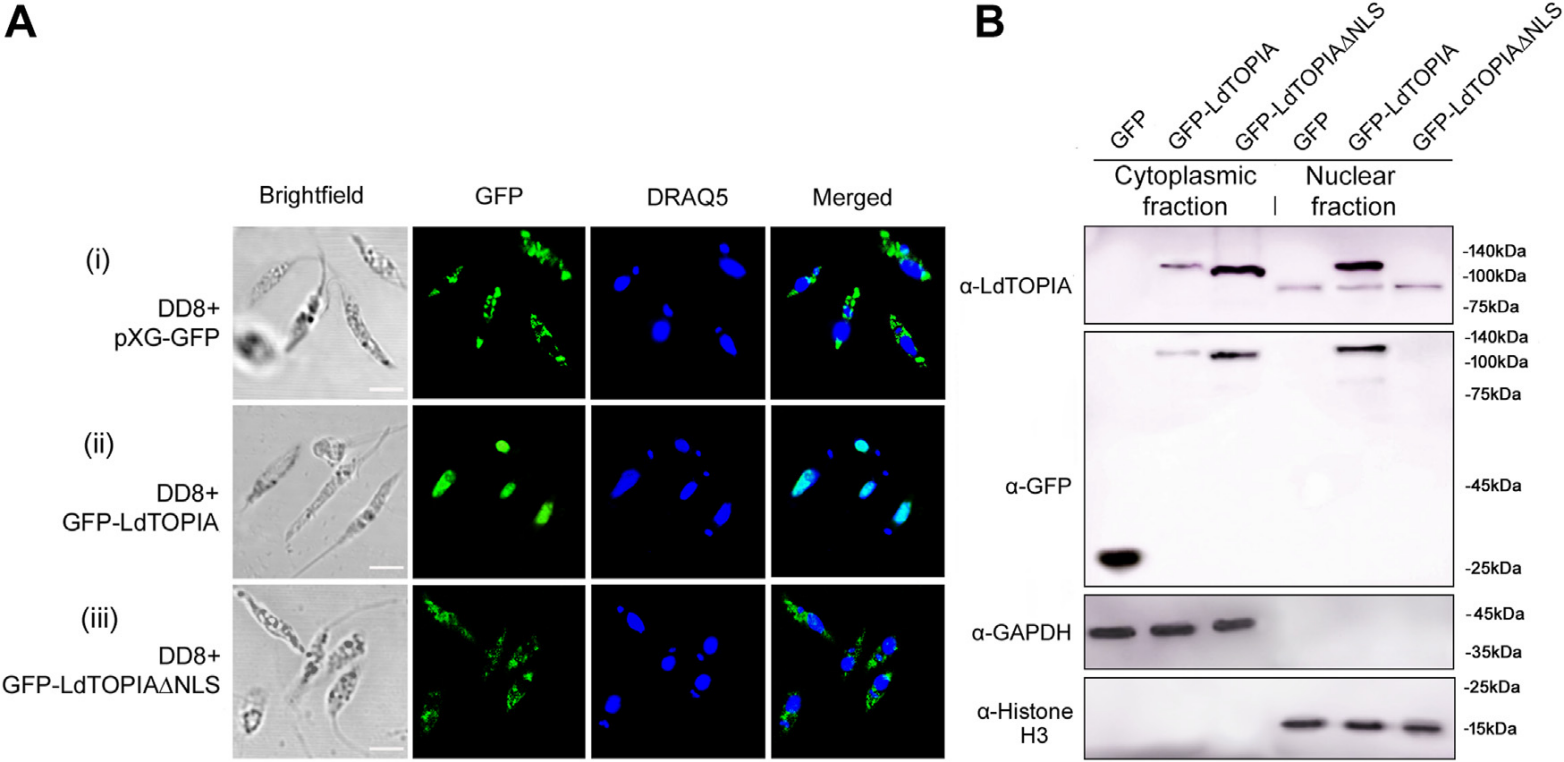
**Localization of LdTOPIA.***A*, microscopic images of *L. donovani* DD8 transfected with pXG-GFP, pXG-GFP-LdTOPIA and pXG-GFP-LdTOPIAΔNLS. Brightfield; GFP: Green fluorescence of GFP or GFP fusion proteins. The parasites were counterstained with DRAQ5 to stain the nucleus and kinetoplast. Scale bar: 5 μm. *B*, immunoblotting of 30 μg cytoplasmic and nuclear extracts prepared from pXG-GFP, pXG-GFP-LdTOPIA, and pXG-GFP-LdTOPIAΔNLS transfected *L. donovani* DD8 parasites using anti-LdTOPIA and anti-GFP antibodies. Anti-GAPDH and anti-Histone H3 were used as cytoplasmic and nucleus-specific loading controls, respectively.

Motif scan analysis of LdTOPIA revealed two bipartite nuclear localization signals (NLS) ^782^KKESHGAACKVDKKTPRRFRAKPKKPKK^809^ at the C-terminus of LdTOPIA. To analyze the precise localization of LdTOPIA inside the parasites, it was tagged at the N-terminus with GFP. It was observed that while the empty vector-transfected parasites exhibited a cytoplasmic fluorescence corresponding to GFP (Fig. 2*A-*i), GFP-LdTOPIA exhibited nuclear fluorescence (Fig. 2*A-*ii). Next, when these NLS sequences between the residues 782aa to 809aa were deleted to generate the GFP-LdTOPIAΔNLS construct and transfected into the parasites, it exhibited cytoplasmic localization (Fig. 2*A-*iii). This indicated that the Motif scan predicted C-terminal NLS of LdTOPIA were functional.

Next, cytoplasmic and nuclear fractions prepared from the above-mentioned cells were immunoblotted to confirm the nuclear localization of LdTOPIA. Immunoblotting with anti-LdTOPIA antibodies exhibited bands corresponding to endogenous LdTOPIA in all three transfectant parasites’ nuclear extracts but not in their cytoplasmic extracts (Fig. 2*B*). Bands corresponding to GFP-LdTOPIA was predominantly observed in the nuclear fraction while slight bands were observed in cytoplasmic fractions that could be the result of extra-chromosomal cytoplasmic overexpression of GFP-LdTOPIA that fails to translocate to the nucleus. The bands for slightly smaller NLS deleted GFP-LdTOPIAΔNLS construct were observed only in the cytoplasmic extracts. Simultaneously immunoblotting of the same fractions using anti-GFP exhibited bands corresponding to empty vector encoded GFP in the cytoplasmic fraction and GFP-LdTOPIA was predominantly present in the nuclear extract than cytoplasm as was observed in anti-LdTOPIA blots. GFP-LdTOPIAΔNLS specific band was observed only in the cytoplasmic extract (Fig. 2*B*). The equal band intensities of GAPDH only from the cytoplasmic fraction and Histone H3 only from the nuclear fraction serve both as loading control and control for fractionation efficiency. These data confirmed that LdTOPIA is localized specifically in the nucleus and deletion of the C-terminal nuclear localization signals makes it localize in the cytoplasm.

Further localization studies were carried out where GFP-LdTOPIAΔNLS was tagged in the C-terminus with an SV40 T-antigen NLS sequence. This construct when transfected into the parasite was relocated back to the nucleus from the cytoplasm (Fig. S2*A-*i). This clearly indicated that the C-terminal residues KKT-PRR and PKK-PKK of LdTOPIA are functional NLS sequences which when deleted prevent the protein from localizing to the nucleus. Since LdTOPIA is a prokaryotic TOPIA homolog we next cloned *E. coli* TOPIA into pXG-GFP and transfected it inside *Leishmania*. GFP-EcTOPIA was localized in the cytoplasm, as EcTOPIA being a prokaryotic protein does not harbor any nuclear localization signal (Fig. S2*A-*ii). Next, when the C-terminal residues KKESHGAACKVDKKTPRRFRAKPKKPKK harboring the functional NLS sequence of LdTOPIA was tagged at the C-terminus of EcTOPIA and transfected into *Leishmania* it exhibited nuclear localization (Fig. S2*A-*iii). Additionally, heterologous expression of EGFP-LdTOPIA in mammalian cells also exhibited nuclear localization while the deletion mutant EGFP-LdTOPIAΔNLS, when transfected into *Leishmania*, exhibited cytoplasmic localization (Fig. S2*B*). The addition of an SV40 T antigen NLS sequence to both EGFP or EGFP-LdTOPIAΔNLS resulted in the nuclear localization of these proteins. This conclusively established that the C-terminal KKT-PRR and PKK-PKK of LdTOPIA are functional nuclear localization signals enabling the nuclear localization of LdTOPIA.

### Nuclear LdTOPIA is a functional homolog of prokaryotic TOPIA

**Figure 3:**
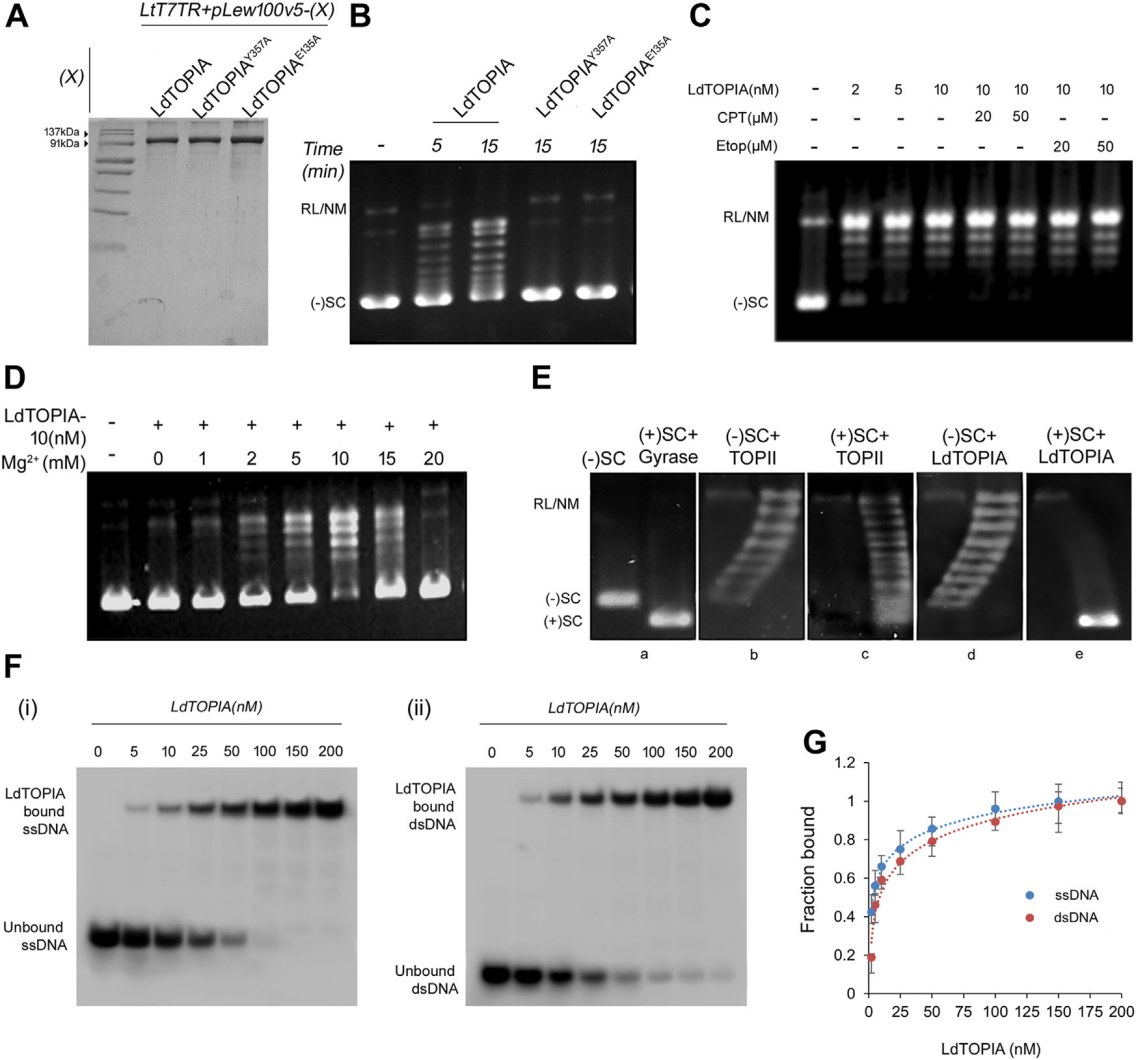
**Functional characterization of purified LdTOPIA.***A*, SDS-PAGE (10%) analysis of purified LdTOPIA, LdTOPIA^Y357A^, and LdTOPIA^E135A^ from tetracycline induced, pLew100v5 cloned LdTOPIA, LdTOPIA^Y357A^, and LdTOPIA^E135A^ transfected *LtT7TR* conditional expression system, stained with Coomassie G-250. Plasmid DNA relaxation assay using (−SC) pBluescript and (*B*) LdTOPIA or its active site mutants LdTOPIA^Y357A^ and LdTOPIA^E135A^ or (*C*) LdTOPIA along with Camptothecin (CPT) or Etoposide (Etop) and in presence of Mg^2+^ at 37 °C for 25 min followed by electrophoresis in 1% agarose gel and thereafter EtBr staining for visualization. *D*, plasmid DNA relaxation assay using (−SC) pBluescript, increasing concentration of Mg^2+^ and purified LdTOPIA at 37 °C for 15 min. *E*, bidirectional agarose gel electrophoresis using (−SC) pBluescript and reverse gyrase generated (+SC) pBluescript plasmid DNA incubated with Human TOPII and purified LdTOPIA in order to differentiate the relaxation of negative and positive topoisomers. *F*, electrophoretic mobility shift assay (EMSA) using 100 nM γ-32P labeled (i) single-stranded and (ii) double-stranded oligonucleotide substrates incubated with increasing concentrations of LdTOPIA (5–200) nM (*G*) DNA binding affinity was measured by fluorescence polarization using 5′ FAM tagged ssDNA and dsDNA substrate incubated with increasing concentration of LdTOPIA. Fraction-bound values were plotted against LdTOPIA concentration (2–200) nM and K_D_ values of LdTOPIA were calculated for ssDNA and dsDNA (n = 5, mean ± SD, 3 biological replicates).

To obtain purified recombinant LdTOPIA we first cloned it in pET28a and transformed it into *E. coli* Rosetta strain, but the overexpressed protein was in the insoluble fraction, which when purified using urea did not yield a functionally active protein. The purified proteins were used to generate antibodies against LdTOPIA. So, we decided to use the *L. tarentolae* protein expression system as per the given schematic (Fig. S3*A*) where LdTOPIA was cloned in pLew100v5 vector and thereafter transfected and expressed through tetracycline induction in the *LtT7TR* strain. Tetracycline-induced transfected parasites were lysed and Ni-NTA affinity chromatography was carried out followed by gel filtration in the S200 column to purify recombinant LdTOPIA to essential homogeneity as shown (Fig. 3*A*). The site-directed active site residue mutants, LdTOPIA^Y357A^ and LdTOPIA^E135A^ were similarly cloned, expressed, and purified to essential homogeneity (Fig. 3*A*).

Overexpressed and purified LdTOPIA when incubated with pBlueScript plasmid DNA, could efficiently relax the plasmid DNA (Fig. 3*B*) as evident from the decrease in the supercoiled band and appearance of the increasing number of intermediate supercoils. The site-directed mutants LdTOPIA^Y357A^, and LdTOPIA^E135A^ failed to relax the plasmid DNA. This indicated that LdTOPIA is a functional protein where Y357 is the active site tyrosine and similar to the prokaryotic TOPIA, the E135 residue also plays a crucial role in DNA relaxation. A multiple sequence alignment of different TOPIA sequences indicated the consensus sequence (DXDREGE) and conserved active site sequence (GYITYPRTDST) for all the protein sequences and that LdTOPIA harbors the E135 and Y357 residues among these conserved sequences, respectively (Fig. S3*B*). LdTOPIA could exhibit plasmid DNA relaxation in the presence of Camptothecin (CPT) as well as Etoposide (Fig. 3*C*) thus further indicating that it is a Type IA topoisomerase and the exhibited DNA relaxation was not due to any TOPIB or TOPII enzymes as Etoposide and CPT could effectively inhibit plasmid relaxation of the TOPIB and TOPII respectively (Fig. S3*C*). Further LdTOPIA also exhibited Mg^2+^-dependent DNA relaxation activity (Fig. 3*D*) which is another characteristic of Type IA topoisomerase activity.

When plasmid relaxation was carried out in the presence of increasing (Na^+^) salt concentration optimal plasmid relaxation was observed at 100 mM of salt concentration (Fig. S3*D*). Type IA topoisomerases can specifically relax only negatively supercoiled DNA. To distinguish between intermediate negative and positive supercoiled topoisomers, the reaction mixtures were electrophoresed in an agarose gel and thereafter at 90° rotation in the presence of chloroquine, a DNA intercalator. *E. coli* DNA gyrase not only can relax both negative and positive supercoils but it can also introduce negative supercoils. Whereas human TOPII can relax both negative and positive supercoils but do not introduce negative supercoils. Hence, when (+) SC DNA was incubated with DNA gyrase it could relax it and introduced negative supercoils which when run in one direction gel electrophoresis in presence of chloroquine, migrated ahead of (−) SC DNA. The compactness of negatively supercoiled DNA is less compared to its positively supercoiled counterpart and thus it runs ahead of negative supercoiled DNA in an agarose gel (Fig. 3*E-*a). On the other hand, the completely relaxed band generated out of human TOPII incubation with (−) SC DNA, in the presence of chloroquine migrated fastest than the nicked DNA and other underwound intermediate negative supercoils migrate as an arc in second dimension (Fig. 3*E-*b and c). Whereas relaxed intermediate supercoils generated by TOPII with (+) SC, which contain positive supercoils, could not absorb DNA intercalator and thus there is a slight change in migration observed based on differences in superhelicity. We found similar results with LdTOPIA and (−) SC DNA as with TOPII suggesting that it can only relax (−) SC DNA but cannot introduce negative supercoils. Also, LdTOPIA could not relax (+) SC DNA (Fig. 3*E-*d and e) which is a property of Type IA topoisomerases.

To analyze the DNA binding ability of LdTOPIA we carried out EMSA analysis. Increasing concentrations of LdTOPIA were incubated with a similar amount of γ-32P end-labeled single-stranded and double-stranded DNA substrates similar to that used for analyzing the DNA binding of *E. coli* TOPIA. It was observed that for the same concentration of LdTOPIA reduced amount of unbound single-stranded substrate (Fig. 3*F-*i) was present in comparison to the double-stranded substrate (Fig. 3*F-*ii). A graph from anisotropy data of bound oligo for single and double-stranded substrates against respective LdTOPIA concentration was plotted in a curve (Fig. 3*G*) from which the K_D_ value of LdTOPIA for single and double-stranded DNA was observed to be 3.16 ± 0.12 nM and 8.1 ± 0.23 nM respectively. This indicates that LdTOPIA has a greater affinity for single-stranded substrates, which is another characteristic of Type IA topoisomerases. We also performed a fluorescence anisotropy analysis using LdTOPIA and human catalytically inactive RNaseH I along with DNA: RNA duplex. The RNA oligomer of the DNA: RNA duplex used in the study was 5′FAM tagged while the DNA sequence was unlabeled and complementary to this RNA sequence. While catalytically inactive human RNaseH I (24) could effectively bind to this DNA: RNA duplex, no binding was observed for LdTOPIA suggesting LdTOPIA has no binding affinity for DNA: RNA duplex (Fig. S3*E*).

### Nuclear LdTOPIA can functionally complement *E. coli* TOPIA null mutant strain

**Figure 4:**
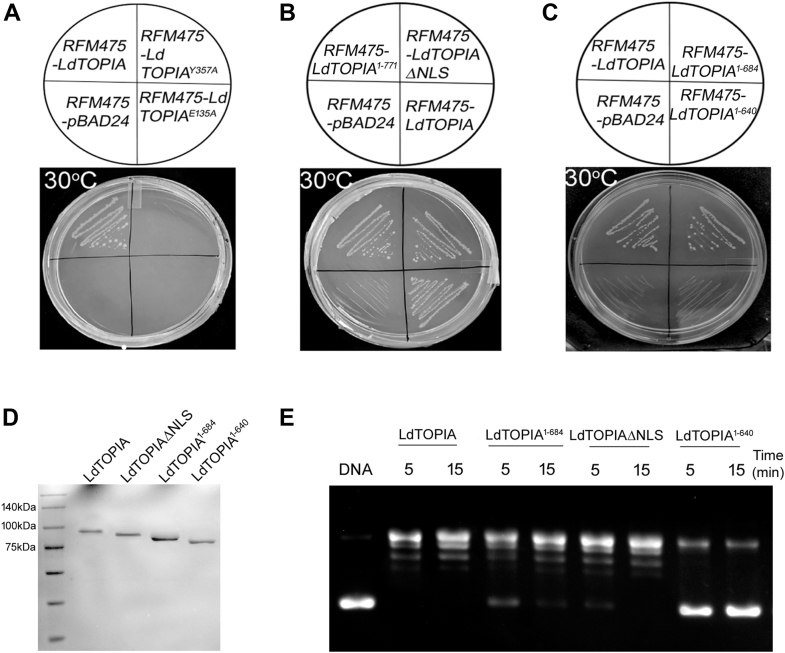
**LdTOPIA can functionally complement the RFM475 (*E. coli* TOPIA null GyrB ts) strain.***A*-*C*, To LB media agar plates containing ampicillin, the following strains RFM475 transformed with pBAD24, pBAD24-LdTOPIA were streaked and grown at non-permissible temperature 30 °C for 36 h. *D*, SDS-PAGE (10%) analysis of purified LdTOPIA and its deletion mutants stained with Coomassie G-250. *E*, plasmid relaxation assay using 10 nM of each purified protein LdTOPIA, LdTOPIAΔNLS, LdTOPIA^1-684^, LdTOPIA^1-640^ at 37 °C.

From the above experiments, it is evident that LdTOPIA is a prokaryotic TOPIA homolog, hence we wanted to check if it could functionally complement *E. coli* TOPIA. For this we took a TOPIA null GyrB ts strain RFM475, kindly gifted by Prof. Marc Drolet along with the control strain RFM445. RFM475 strain could grow slowly at 37 °C as DNA gyrase could partly complement the functionality of TOPIA but at non-permissive temperatures of 30 °C and 42 °C where GyrB ts becomes non-functional, thus having no active topoisomerase, which then would lead to growth lethality (8). LdTOPIA as well as the active site mutants, LdTOPIA^Y357A^ and LdTOPIA^E135A^ were cloned in the arabinose inducible pBAD24 vector and transformed into RFM475 strain. LdTOPIA expressing RFM475 transformants could only grow at the non-permissive temperature of 30 °C as well as 42 °C (Fig. S4*A*) indicating thereby that LdTOPIA could functionally complement *E. coli* TOPIA null mutant. Growth kinetics of LdTOPIA expressing RFM475 along with RFM475 alone or expressing pBAD24 or RFM445 strains, carried out at 30 °C also exhibited a similar result (Fig. S4*B*). But RFM475 alone or LdTOPIA^Y357A^ and LdTOPIA^E135A^ expressing transformants failed to grow at 30 °C (Fig. 4*A*) and the same was also evident in the growth kinetic study as shown in (Fig. S4*C*). This indicated that wild-type LdTOPIA and not its active site mutants could functionally complement the growth lethality of the RFM475 strain at non-permissive temperatures.

Compartmentalization in eukaryotes makes NLS signals essential for the proper functionality of LdTOPIA. Since prokaryotes do not exhibit such compartmentalization, we next wanted to check if deletion of the NLS sequences from LdTOPIA could still make it complement *E. coli* TOPIA in the RFM475 strain. LdTOPIAΔNLS as well as LdTOPIA^1-771^, when transformed into RFM475 strain, exhibited growth at the non-permissive temperature of 30 °C (Figs. 4*B* and S4*D*), indicating thereby that deletion of the NLS sequences does not hamper its topoisomerase activity. With respect to prokaryotic TOPIA the residues crucial for DNA binding were present at the C-terminus followed by a zinc finger domain. Although lacking a Zn-finger domain, similar residues were harbored between amino acids 640 to 684 of LdTOPIA. Hence, we generated the deletion constructs LdTOPIA^1-684^ and LdTopIA^1-640,^ cloned it in pBAD24, and transformed into RFM475 strain. LdTOPIA^1-640^ failed to complement while LdTOPIA^1-684^ although exhibited reduced growth could still complement TOPIA null and GyrB ts RFM475 at 30 °C indicating thereby that the residues between 640 to 684 although lacking Zn-finger domain as in *E. coli* TOPIA harbors residues crucial for exhibiting topoisomerase activity (Fig. 4*C*). Growth kinetics of the complementing LdTOPIAΔNLS and LdTOPIA^1-640^ deletion mutants where the former exhibited growth at 30 °C and the later failed to grow at 30 °C were also obtained (Fig. S4*E*). From these data it is evident that LdTOPIA being a homolog of prokaryotic TOPIA could functionally complement it in RFM475 strain and the same is prevented if the active site residues are mutated as in LdTOPIA^Y357A^ and LdTOPIA-^E13^^5^^A^ or DNA binding regions are deleted as in LdTOPIA^1-640^. Besides, the NLS sequences of LdTOPIA are not required for functional complementation in RFM475 strain. We next purified the NLS deleted LdTOPIAΔNLS (1-781aa), LdTOPIA^1-684^, LdTOPIA^1-640^ along with wildtype LdTOPIA (809aa) and electrophoresed them in an SDS PAGE (Fig. 4*D*). Thereafter, we carried out a DNA relaxation assay using these purified proteins (Fig. 4*E*). It was observed that deletion of the 782-809aa (NLS), and 685-809aa (LdTOPIA^1-684^) had negligible effect on the DNA relaxation as compared to LdTOPIA and therefore they could also complement EcTOPIA in RFM475 strain while the 641-809aa deleted construct LdTOPIA^1-640^ failed to relax supercoiled DNA, hence it had failed to complement EcTOPIA in RFM475 strain. The unusual bi-subunit TOPIB of *Leishmania* (LdTOPIL/S) (25, 26) is a crucial enzyme of *Leishmania* that can also relax negative supercoils. From literature we know that when the small subunit (LdTOPS) is fused in tandem with the large subunit (LdTOPIL) and expressed and purified as a single protein as LdTOPIL-*fus*-S it exhibits properties similar to the monomeric human TOPIB (26). To analyze whether *Leishmania* TOPIB (LdTOPIL-*fus*-S) has an effect on the R-loop resolving/preventing process we did complementation assays in RFM475 strain. LdTOPIL-*fus*-S could not complement *E. coli* TOPIA as was done by LdTOPIA (Fig. S4, *F* and *G*).

### Nuclear R-loops, formed during polycistronic transcription, are resolved by LdTOPIA

Prokaryotic TOPIA is known to prevent R-loop formation during polycistronic prokaryotic transcription. Since lower eukaryote *Leishmania* harbors a near intron-less genome, transcription in *Leishmania* is also said to be polycistronic, thus posing a problem of R-loop formation, if not prevented. The presence of spliced-leader RNA required for trans-splicing provides further evidence towards initial polycistronic transcription and later separate mRNA production through trans-splicing (1). The presence of a functionally complementing prokaryotic TOPIA homolog in *Leishmania* which could functionally complement it, made us assume that LdTOPIA could have been retained in *Leishmania* to play a similar role in preventing R-loops.

**Figure 5:**
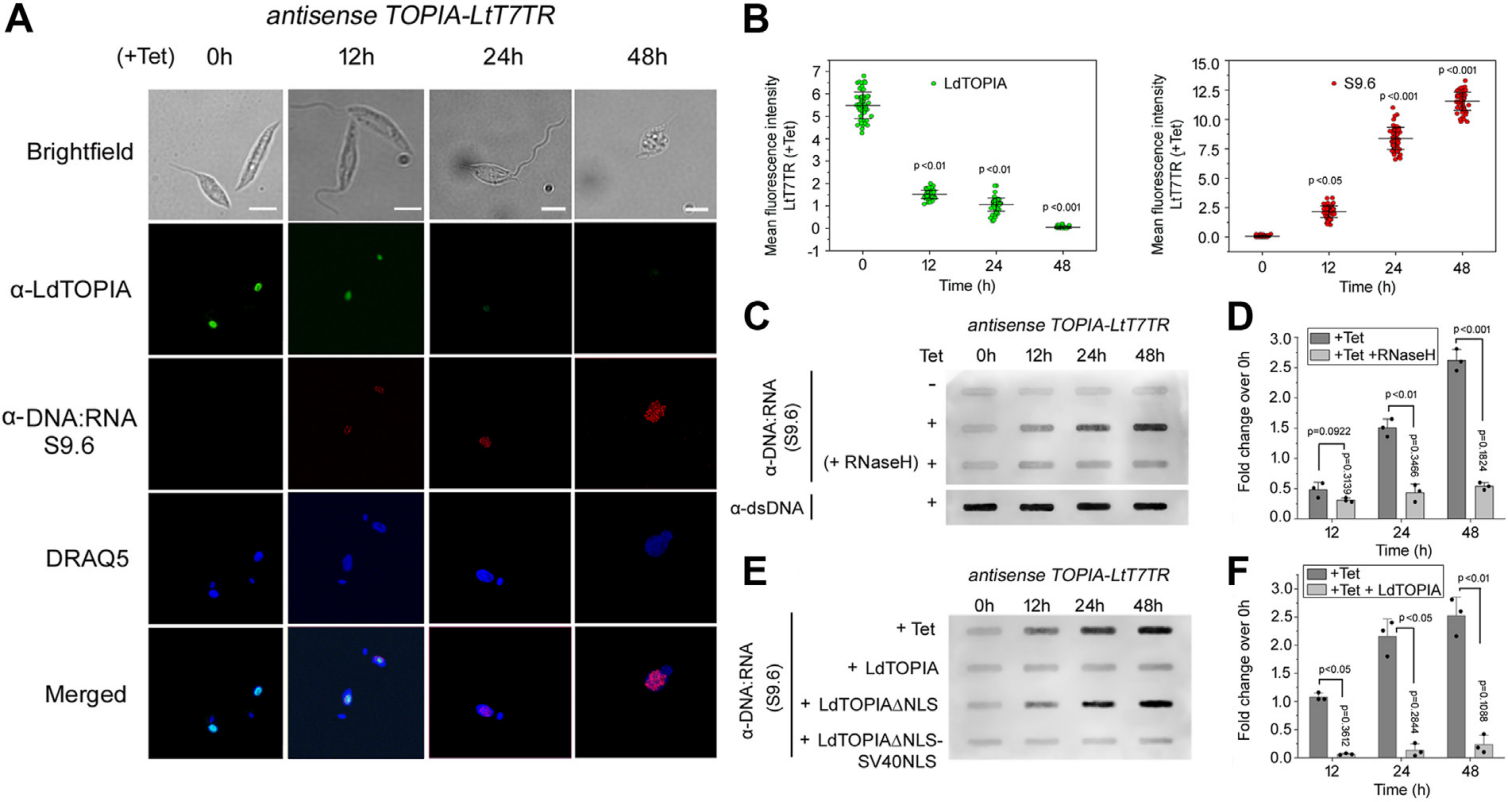
**LdTOPIA prevents R-loop formation during polycistronic transcription.***A*, LtT7TR strain transfected with anti-LtTOPIA construct was either untreated or treated with tetracycline for indicated time points to observe the extent of R-loop formation. Parasites were stained with anti-LdTOPIA-AlexaFluor488, anti-DNA-RNA (S9.6) Alexa Fluor568 antibodies, and counterstained with DRAQ5. Scale Bar, 5 μm. *B*, graphical representation of the extent of R-loop formation estimated from the fluorescence intensity. [n = 60 (20 nuclei of 3 biological replicates), mean ± SD. p *versus* 0h]. *C*, DRIB assay was carried out from genomic DNA isolated from untreated or tetracycline-treated antisense LtTOPIA transfected LtT7TR parasites after indicated time points. *D*, densitometry of the same samples. [n = 3 and 3 biological replicates, mean ± SD, *p* values for (+Tet) *versus* (+Tet +RNaseH) and p *versus* 0h of (+Tet +RNaseH)]. *E*, DRIB assay was carried out using genomic DNA isolated from untreated or tetracycline-treated antisense LtTOPIA transfected LtT7TR parasites and complemented with LdTOPIA, LdTOPIAΔNLS or LdTOPIAΔNLS-SV40NLS for indicated time points. *F*, densitometric analysis of the same samples [n = 3 and 3 biological replicates, mean ± SD, *p* values for (+Tet) *versus* (+Tet +LdTOPIA) and p *versus* 0h of (+Tet +LdTOPIA)].

To check the formation of R-loops inside the parasite nucleus, we used the conditional antisense-mediated LtTOPIA knockdown strain (antiTOPIA). Parasites when treated with tetracycline induced the downregulation of LtTOPIA and the cells were thereafter stained with anti-LdTOPIA and anti-DNA-RNA hybrid antibody (S9.6) for different timepoints. The punctate stain corresponding to DNA-RNA hybrids or R-loops increased with increasing time of tetracycline treatment inside the parasite nucleus observed (Fig. 5*A*). Quantitative analysis of the same showed an increase in R-loop formation with a decrease in LtTOPIA with time (Fig. 5*B*). Further, we also performed a DRIB assay, wherein after the indicated time of tetracycline induction of antisense constructs in the parasites, genomic DNA was isolated and dot-blotted using an anti-DNA-RNA hybrid antibody. It was observed that the amount of DNA-RNA hybrids present in the genomic DNA, increased with increased time of tet induction (corresponds to rapid reduction of LtTOPIA), while no DNA-RNA hybrids were observed in the tet (−) parasites (Fig. 5*C*). Densitometric analysis shows that compared to control loss of LdTOPIA resulted in a 2.6-fold higher amount of R-loop formation after 48 h of tet induction. The increasing intensity in the dot blots with rapid reduction of LtTOPIA disappeared and it became similar to control levels when the isolated genomic DNA was treated with RNaseH prior to dot blot analysis (Fig. 5, *C* and *D*). This indicated clearly that the increasing intensity of the dot blots was due to increasing amounts of R-loop formation due to loss of LtTOPIA.

Since the anti-sense construct was from the 5′UTR region of endogenous LtTOPIA we next wanted to carry out complementation studies. For this, we transfected the antisense transfectant with wildtype and different mutants of LdTOPIA. With increasing time of tetracycline induction of antisense of endogenous LtTOPIA, wild-type LdTOPIA could complement the endogenous protein and thereby prevent R-loop or DNA-RNA hybrid formation (Fig. 5, *E* and *F*). But antiTOPIA vector control alone or the NLS deleted construct of LdTOPIA exhibited increasing amounts of R-loop formation with time since the former transfectants had no functional LdTOPIA, while the later although having a functional LdTOPIA could not localize to the nucleus to prevent R-loop formation during polycistronic transcription. Therefore, when we next added an SV40 T-antigen NLS sequence to the C-term of LdTOPIAΔNLS (LdTOPIAΔNLS-SV40NLS), no R-loop formation was observed with increasing time of tetracycline induction owing to re-localization of LdTOPIAΔNLS-SV40NLS to the nucleus (Fig. 5*E*). These data clearly indicated that LdTOPIA being a nuclear protein can resolve polycistronic transcription generated R-loops inside the parasite.

We observed earlier that LdTOPIL-*fus*-S could not complement *E. coli* TOPIA in the RFM475 strain. Moreover, endogenous *Leishmania* TOPIB (LtTOPIL/S) failed to complement the loss of LtTOPIA upon its tetracycline-induced conditional antisense. Yet we carried out a complementation study wherein we cloned LdTOPIL-*fus*-S (26) in pXG vector and transfected it in the LtTOPIA conditionally antisense LtT7TR strain (antiTOPIA). The addition of tetracycline for increasing timepoints exhibited increased amounts of DNA: RNA hybrids which was not evident for similar parasites transfected with LdTOPIA (Fig. S5, *A-*i and *A-*ii). This further indicated that LdTOPIL-*fus*-S cannot prevent R-loop formation.

From the literature, it is evident that RNaseH IIA of *Trypanosoma* is localized in the nucleus where it resolves R-loops and does not have any activity in kinetoplast (27). On the other hand, we observed that its homolog RNaseH IIA of *Leishmania donovani* (LdRNaseH IIA) lacks a functional NLS and localizes in the kinetoplast (Fig. S5*B*). To analyze whether LdRNaseH IIA can complement the function of LdTOPIA we cloned LdRNaseH IIA in pXG vector and transfected it in the LtTOPIA conditionally antisense LtT7TR strain (antiTOPIA). The G418 selected transfectant parasites when treated with tetracycline (resulting in antisense of LtTOPIA) for increasing time points resulted in increased DNA: RNA hybrids of the transfectants. Both empty vector (pLew100) and LdRNaseH IIA transfectant exhibited similar extents of DNA: RNA hybrids (Fig. S5, *C-*i and *C-*ii). This indicated that LdRNaseH IIA cannot complement LtTOPIA (or similarly LdTOPIA).

### Tricyclic anti-depressant norclomipramine by inhibiting LdTOPIA generates R-loops which leads to parasite elimination

**Figure 6:**
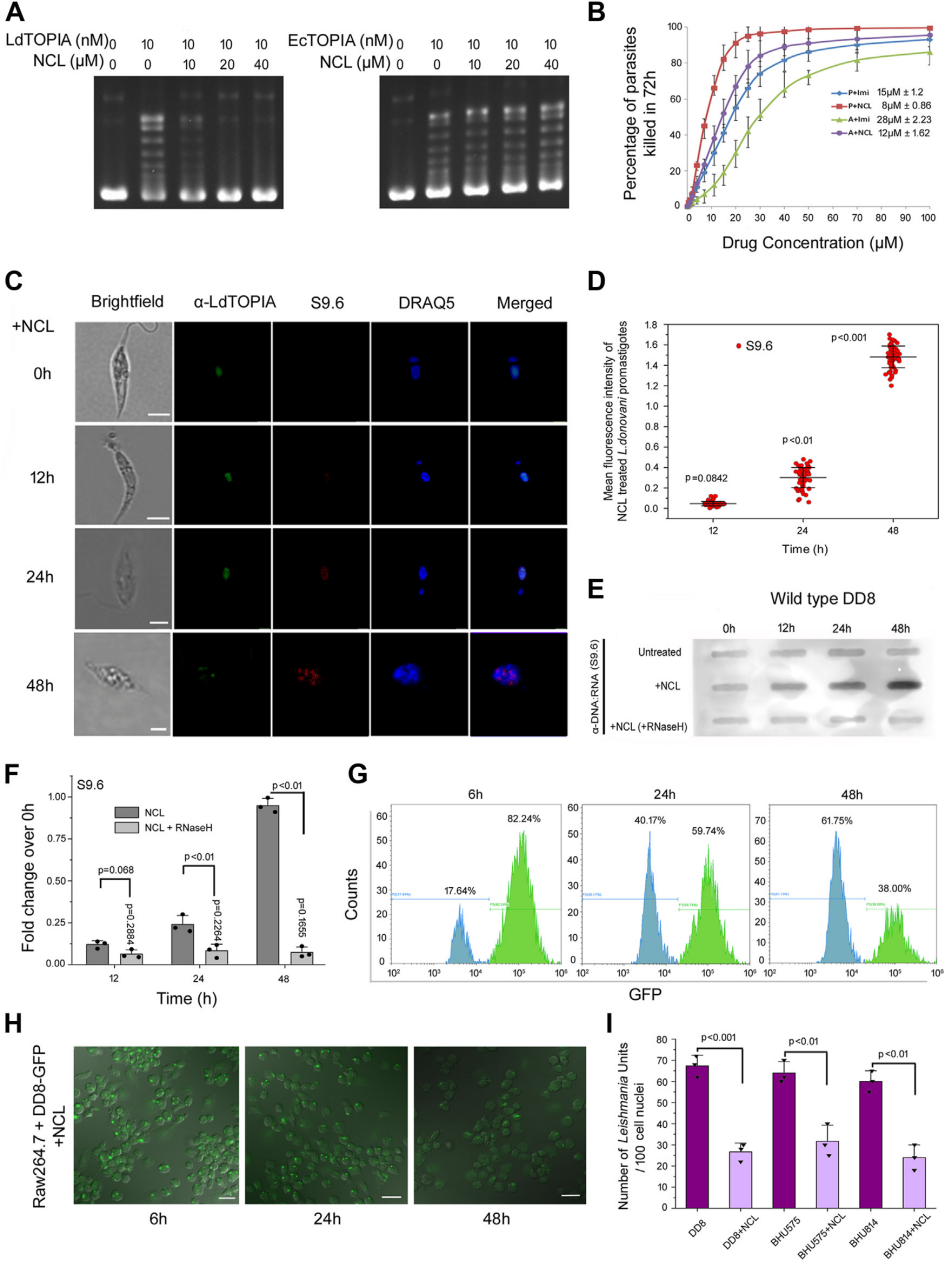
**Inhibition of LdTOPIA by norclomipramine leads to parasite elimination.***A*, DNA relaxation assay was carried out using (−) SC pBluescript DNA, purified (*left panel*) LdTOPIA (10 nM) and (*right panel*) EcTOPIA (10 nM) and in absence or presence of increasing concentration of norclomipramine. *B*, modified MTT assay using increasing concentration of norclomipramine or imipramine treated *L. donovani* DD8 parasites promastigotes and axenic amastigotes to monitor percentage of parasite killing. IC50 values were calculated from the graph plotted, percentage of parasites killed *versus* NCL/Imi concentration. (n = 3 and 3 biological replicates, mean ± SD). *C*, immunofluorescence analysis of 10 μM NCL-treated *L*. *donovani* DD8 to analyze R-loop formation. Parasites upon treatment for indicated timepoints were fixed and incubated with anti-LdTOPIA-AlexaFluor488, anti-DNA-RNA hybrid (S9.6)-AlexaFluor568 antibodies, and counterstained with DRAQ5. Scale Bar, 5 μm. *D*, graphical representation of the extent of R-loop formation estimated from the fluorescence intensity. [n = 60 (20 nuclei of 3 biological replicates), mean ± SD. p *versus* 0h]. *E*, DRIB assay was carried out from untreated and NCL treated, genomic DNA isolated and RNaseH treated samples and (*F*) densitometry of the same samples (n = 3 and 3 biological replicates, mean ± SD). *G*, flow cytometry of Raw264.7 infected with DD8-GFP treated with 10 μM norclomipramine. Graph showing the percentage of infected macrophages at each time point. *H*, microscopic images of the same. Scale Bar, 20 μm. *I*, intracellular parasite burden, 48 h treatment post-infection with DD8, BHU575 and BHU814. (n = 100 cell nuclei, values averaged and plotted for 3 biological replicates, mean ± SD).

Tricyclic anti-depressants (TCA) had previously been shown to exhibit anti-leishmanial activity and another study also showed that imipramine can stimulate IL-10 production and promote parasite killing. In another study, mycobacterial topoisomerase IA specifically and not *E. coli* TOPIA was shown to be efficiently inhibited by norclomipramine which is a widely used TCA in humans (28, 29, 30, 31). Both imipramine and norclomipramine (NCL) could inhibit the DNA relaxation by LdTOPIA, but NCL was found to be a more potent inhibitor of LdTOPIA as compared to imipramine (Fig. S6*A*). LdTOPIA was closer to mycobacterial TOPIA hence NCL could potently inhibit its DNA relaxation. Next, when we carried out a DNA relaxation assay using LdTOPIA and EcTOPIA in the presence of an increasing concentration of NCL, it was observed that NCL failed to inhibit the DNA relaxation by EcTOPIA (Fig. 6*A*).

Since NCL could effectively inhibit LdTOPIA we wanted to see if this inhibition was due to binding of NCL to the active site or elsewhere. When NCL was docked to the energy-minimized homology-modeled structure of LdTOPIA it was observed that NCL localized to the active site of LdTOPIA (Fig. S6*B*), thus indicating that it is a competitive inhibitor of LdTOPIA. We next wanted to check the anti-leishmanial potential of NCL in comparison to imipramine, as it was a competitive inhibitor of LdTOPIA, an essential enzyme for *Leishmania*. NCL could efficiently eliminate *Leishmania* promastigotes and axenic amastigotes with an IC50 value of 8 μM and 12 μM respectively as compared to 15 μM and 28 μM respectively for imipramine (Fig. 6*B*). By growth complementation study we found that LdTOPIA could complement EcTOPIA in the RFM475 strain, but EcTOPIA was not inhibited by NCL. Hence, further complementation experiments were carried out wherein RFM475 strains were transfected with LdTOPIA, LdTOPIA1-771, and EcTOPIA and grown in arabinose and NCL-containing plates at 30 °C. LdTOPIA and LdTOP1A1-771 which was previously observed to complement EcTOPIA at a non-permissive temperature of 30 °C failed to do so in the presence of NCL, but EcTOPIA could complement the TOPIA null GyrB ts RFM475 strain as it was not inhibited by NCL (Fig. S6*C*). Next with increasing time of NCL treatment of *Leishmania,* there were increasing amounts of R-loops, generated inside the nucleus visualized under the microscope (Fig. 6, *C* and *D*), and the same was also observed by DRIB assay (Fig. 6, *E* and *F*). The NCL treatment generated, increasing amounts of R-loops formed inside the parasite's nucleus was resolved upon treatment of isolated genomic DNA with RNaseH prior to dot-blot analysis (Fig. 6*E*). Since LdTOPIA complements the function in RFM475, we wanted to check if EcTOPIA could complement LdTOPIA and prevent parasite elimination by resolving the R-loops generated by NCL-mediated inhibition of LdTOPIA. But EcTOPIA being a prokaryotic protein lacked NLS. Therefore, we tagged the NLS of LdTOPIA to the C-terminus of EcTOPIA and the construct was transfected into *Leishmania* followed by treatment with NCL. It was observed that EcTOPIA alone could not prevent nuclear R-loop formation due to inhibition of LdTOPIA by NCL as it failed to localize to the nucleus as had been observed earlier. But, EcTOPIA-LdNLS could prevent NCL treatment-generated R-loop formation inside the parasite (Fig. S6, *D* and *E*). Thus, it was clearly established that LdTOPIA is a eukaryotic homolog of prokaryotic TOPIA but adapted for eukaryotic compartmentalization in having a functional NLS so as to localize to the nucleus and prevent any polycistronic transcriptional aftermath generated R-loops inside the parasite.

Further to check the potential of the clinically used FDA-approved tricyclic antidepressant norclomipramine in drug repurposing we next studied whether it could eliminate the clinically isolated antimony-resistant *Leishmania* isolates BHU575 and BHU814. It was observed that NCL could efficiently eliminate both the clinical isolate BHU575 and BHU814 with IC50 values of (11.7 μM ± 0.6) and (10.37 μM ± 0.42), respectively, which was close to that of DD8 parasites (8 μM ± 0.86) (Fig. S6*F*). Moreover, cytotoxicity of NCL upon macrophages (Raw264.7 and THP1) exhibited IC50 values (38.24 μM ± 1.8) and (40 μM ± 2.4) respectively which are ∼5 fold higher than IC50 values for the *Leishmania* (DD8) promastigotes (Fig. S6*G*). Next *L*. *donovani* DD8-GFP infected macrophages were sorted (using GFP marker) and thereafter treated with 10 μM NCL for increasing time points. The decreased number of GFP-positive macrophages with increasing time of NCL treatment as measured through flow cytometry (Fig. 6*G*) or microscopic analysis (Fig. 6*H*) indicated the effective elimination of intracellular amastigotes by NCL. Additionally, macrophages were infected with wild-type DD8 or clinical isolates BHU575 and BHU814 followed by 10 μM NCL treatment for 48 h and thereafter Giemsa stained. Microscopic analysis followed by quantitation of the intracellular amastigotes exhibited a considerably reduced number of intracellular amastigotes in the NCL-treated and different *Leishmania*-infected macrophages as compared to the untreated and infected controls (Fig. 6*I*). These data clearly exhibit the potential of norclomipramine as an effective anti-leishmanial.

## Discussion

Evolutionarily topoisomerases have evolved from primitive organisms to resolve DNA topological tangles formed during essential eukaryotic functions. The intron-less prokaryotes by virtue of exhibiting polycistronic transcription, have the propensity to generate R-loops if not prevented by the presence of Topoisomerase IA (8, 12). The near intron-less trypanosomatids also exhibit polycistronic transcription thus generating a propensity of nuclear R-loop formation prior to its trans-splicing mediated processing and therefore harbors the prokaryotic TOPIA homolog in its genome. Introns are known to play a critical role in preventing R-loop mediated deleterious DNA damage hence we were skeptical about the presence of R-loop in near intron-less lower eukaryotes like *Leishmania*. Higher eukaryotes by virtue of having introns exhibit considerably reduced propensity for R-loop formation (17, 18). While Topoisomerase IA is known to prevent R-loop formation, RNaseH can resolve R-loops or DNA-RNA hybrids (32, 33, 34). Interestingly RNaseH IIA has been shown to resolve nuclear R-loops in *Trypanosoma* since the TOPIA homolog which lacked a functional NLS made it localize to the kinetoplast to resolve theta structures during minicircle replication (6, 27). Contrary to this we observed that LdTOPIA by virtue of having functional NLS localizes to the nucleus of *Leishmania* where it helps prevent R-loop formation. Moreover, RNaseH IIA of *Leishmania* which lacks an NLS sequence localizes to the kinetoplast of these parasites. This indicates that with respect to DNA Topoisomerase IA and RNaseH IIA, *Trypanosoma* and *Leishmania* exhibit role reversal. *Leishmania donovani* being an old-world kinetoplastid (35) and closer to prokaryotes, harbor a nuclear DNA Topoisomerase IA involved in preventing R-loop formation during polycistronic transcription while *Trypanosoma brucei* is a new world kinetoplastid (36) and harboring mobile Type II intron in its genome have localized DNA Topoisomerase IA to the kinetoplast and can resolve R-loops if generated through RNaseH IIA similar to higher eukaryotes. The evolutionary functional diversity between two close organisms exhibits gradual loss and gain of function during divergence.

**Figure 7:**
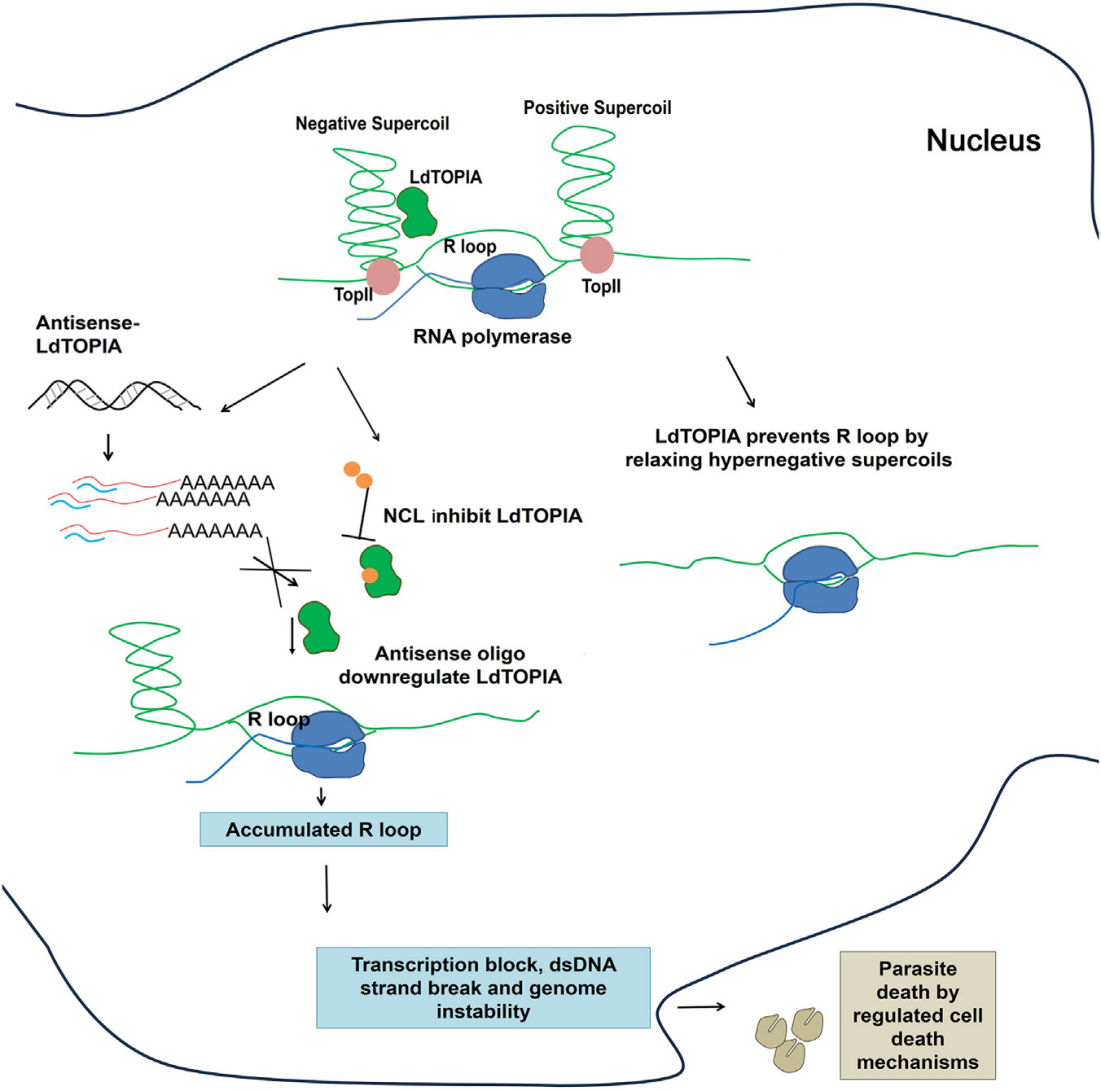
**Schematic representation on the functional role of LdTOPIA in preventing nuclear R-loops inside the parasite nucleus and the effect of conditional antisense-mediated downregulation of LdTOPIA or norclomipramine mediated inhibition of LdTOPIA to induce R-loop mediated genome instability and parasite elimination**.

A conditional knockdown of *Leishmania* TOPIA resulted in serious growth defects and cell cycle arrest at G2/M phase thereby suggesting that LdTOPIA is an essential enzyme for *Leishmania*. LdTOPIA is not only a close homolog of prokaryotic TOPIA but it also retains the activity of resolving hypernegative supercoils. Similar to prokaryotic TOPIA, we found LdTOPIA could only relax negatively supercoiled DNA strictly in the presence of Mg^2+^ and has a higher affinity toward single-strand DNA substrate. On the other hand, active site mutants could not retain the activity. Similar results were obtained where wild-type and NLS-deleted LdTOPIA could complement the function of TOPIA null mutant, RFM475 strain whereas the active site mutants were unable to do the same. These further confirmed that *Leishmania donovani* topoisomerase LdBPK_210180.1 is Type IA topoisomerase. Since *Leishmania* RNaseH II do not harbor NLS and was observed to localize to the kinetoplast and LdTOPIA could complement *E. coli* TOPIA null mutant strain by preventing R-loop formation, we hypothesized that LdTOPIA could be preventing nuclear R-loops that may arise due to polycistronic transcription of intron-less *Leishmania*. Interestingly, LdTOPIA could prevent DNA-RNA hybrid or R-loop formation inside RFM475 strain, and downregulation of the enzyme resulted in the formation of DNA-RNA hybrids inside the parasite. This clearly indicates that LdTOPIA is required to prevent nuclear R-loop formation inside the parasite (Fig. 7). Tricyclic anti-depressants have previously been shown to be effective anti-leishmanials of which imipramine has previously been reported to inhibit trypanothione reductase of *Leishmania* and TOPIA of mycobacteria (37, 38). Norclomipramine was also shown to inhibit mycobacterial TOPIA. In this study, it was observed that norclomipramine was a more potent inhibitor of LdTOPIA than imipramine. Additionally, norclomipramine could not inhibit the DNA relaxation by *E. coli* TOPIA. Thus, it was evident that norclomipramine is a more effective anti-leishmanial through its efficient inhibition of LdTOPIA activity and therein generating R-loops leading to genomic instability and consequent death of the parasites. Moreover, it was observed that expression of NLS-tagged *E. coli* TOPIA inside the parasites could prevent the norclomipramine treatment-induced R-loop formation. The most effective drug Ambisome is not without side effects and it surfaces up as PKDL at a later time point (39, 40). Also developing drug resistance poses a critical situation in the treatment of visceral leishmaniasis (41). In this scenario finding new drug targets and treatment strategies are crucial. Here we revealed the essential function of LdTOPIA which is inhibited by norclomipramine, a clinically established tricyclic antidepressant that at a much lower dose can be repurposed to treat visceral leishmaniasis.

## Experimental procedures

### Microbial strains and cell lines maintenance

*E. coli* DH5α strain used for clone selection. *E. coli* RFM445 and RFM475 were used for growth complementation studies. RFM475 is TOPIA null, GyrB ts. Bacterial strains were cultured in autoclaved 2% Luria-Bertani broth at 37 °C, 130 rpm in an incubator shaker (Scigenics). *Mycobacterium tuberculosis* H37Ra was cultured in 7H9 media (sigma) with 10% OADC supplement [Oleic acid (Sigma), Albumin (SRL), Dextrose (sigma), Catalase (sigma)]. An Indian isolate of *Leishmania donovani* (MHOM/IN/DD8/1968) was maintained in M199 media (sigma), supplemented with 10% Fetal Bovine Serum (Gibco) at 23 °C. For knockdown studies, the *Leishmania tarentolae* (LtT7TR) strain was used. To maintain the genome-integrated T7 RNA polymerase and Tet repressor genes, the parasites were cultured in the presence of nourseothricin and hygromycin (100 μg/ml each, GoldBio). Axenic amastigotes were developed by a continuous culture of DD8 promastigotes in RPMI1640, pH-5.5. Macrophage (RAW264.7) was maintained in DMEM (Sigma) with 10% FBS in a 37 °C CO_2_ incubator.

### Bioinformatic analysis

*L. donovani* and *T. brucei* TOPIA protein sequence obtained from Gene DB (http://www.genedb.org). MtTOPIA sequence obtained from Mycobrowser (epfl.ch). *E. coli*, *Mus musculus*, *Homo sapiens*, *S*. *cerevisiae*, *Arabidopsis thaliana*, *Oryza sativa*, *C. merolae*, vaccinia virus TOPI sequences are obtained from NCBI, Bacteria Ensemble and Plant Ensemble as required. Multiple sequence alignment was carried out by Clustal omega software to obtain prokaryotic TOPIA characteristic conserved consensus and active motif sequences. Nuclear localization signal sequence was identified using Expasy Motif Scan (Motif Scan (sib.swiss). Phylogenetic analysis of proteins TOPIA, TOPIII, and TOPIB from different sources was performed by MEGA11 software.

The input sequences are enlisted as follows: [(*Leishmania donovani TOPIA*/Gene Id- LdBPK_210180.1/Amino acid- 809/Genomic location 35228 - 37657 (+strand), Chromosome 21), (*Leishmania braziliensis* TOPIA/Gene Id- LbrM.21.0170/Amino acids-799), (*T. brucei brucei* TOPIA/Gene Id- Tb927.10.1900/Amino acids- 806), (*M. tuberculosis* TOPIA/Gene Id- Rv3646c/Amino acids- 934), (*E. coli* TOPIA/Gene Id *945862/*Amino acids- 865), (*Entamoeba histolytica* TOPIII *putative/*Gene Id- EHI_038920/Amino acids- 607), (*C. merolae* TOPI (Cyanidioschyzon merolae)/Gene Id- CYME_CMI252C/Amino acids- 1090), (*Acanthamoeba polyphaga* (mimivirus) bacterial type/AY653733.1), (*L. donovani* TopIII/Gene Id- LdBPK_281900.1/Amino acids- 867), (*S. cerevisiae* TOPIB/Gene ID: 854156/Amino acids-769), (*A. thaliana* TOPIα/Gene Id- 835623/UniProtKB identifiers: P30181/Amino acids- 916), *H. sapiens* TOPIB/GeneID:7150/Amino acids- 765), (*M. musculus* TOPIB/Gene ID: 21969/Amino acid-767), (*M. musculus* TOPImt/Gene ID: 72960/Amino acids-593), (*H. sapiens* TOPIII/GenBank: BAA20009.1/Amino acids- 753), (*O. sativa japonica* TOPI *(prokaryotic type), putative*/Gene Id- P0456E06.44/Amino acids- 896), (*A. thaliana* TOP Type IA/Gene ID: 829248/Amino acids- 1284), (*M. musculus* TOPIIIα/GeneID:21975/Amino acids- 1003), (Vaccinia virus (strain Copenhagen) TOPIB/Accession- P68697/Amino acids- 314) ]

### Quantitative PCR analysis

Total RNA was isolated from *Leishmania* cells using TRIzol reagent (Life Technologies) and dissolved in nuclease-free molecular grade water in the presence of RNase A Inhibitor (NEB) and stored at −80 °C. Purity of RNA was checked by 260/280 ratio in a spectrophotometer. Followed by DNase I (Roche) treatment, 1 μg of total RNA was denatured and mixed with 1 mM dNTPs, 1× RT buffer and reverse transcriptase, gene-specific primers as recommended in AMV RT kit (NEB), and incubated at 42 °C for 30 min for cDNA synthesis. cDNA preparation confirmed by PCR. Next for quantitative PCR analysis, 100 ng of cDNA and gene-specific primers were mixed with 1× premixed solution Luna qPCR master mix (NEB) of Taq polymerase, dNTPs, and buffer in a 20 μl reaction volume. The RT-PCR analysis was done in a BioRad RT-PCR machine. SYBR green dye binds to double-stranded DNA. The C_t_ values were calculated as fold change in expression.

### Cloning and subcloning of topoisomerase IA (TOPIA) from *L. donovani*, *E. coli* and *M. tuberculosis* for complementation studies

A 2.43 Kb Topoisomerase IA gene (Gene Id LdBPK_210180.1) from chromosome 21, LdTOPIA was PCR amplified from *Leishmania* donovani (DD8) genomic DNA using forward primer 5′ GGAATTCCATATGTTGCGCCGCAGCGTGCGTG3′ and reverse primer 5′ CCGCTCGAGCTACTTCTTCGGCTTCTTCGGCTT3′ and cloned in pET28a bacterial expression vector at NdeI/XhoI sites. pBAD24 vector does not have 6X-His sequence. So, for the purpose of both growth complementation and purification of LdTOPIA, it was PCR amplified using a 6X-His tag sequence corresponding to pET28a sequence with EcoRI overhang 5′CGGAATTCATGCATCATCATCATCATCAC3′ as forward primer and XbaI containing LdTOPIA reverse primer from pET28a-LdTOPIA construct as template and cloned in pBAD24 at EcoRI/XbaI site. Thereafter, subcloning of deletion constructs, LdTOPIAΔNLS (NLS deleted), LdTOPIA-771, LdTOPIA-684, LdTOPIA-640, was done at NdeI/XbaI sites of the pBAD24-LdTOPIA vector. Wildtype EcTOPIA, and wildtype MtTOPIA were cloned at NdeI/XbaI and EcoRI/KpnI sites respectively with stop codon. For EcTOPIA+ LdNLS (NLS of LdTOPIA), and MtTOPIA+ LdNLS, at first EcTOPIA and MtTOPIA was cloned at NdeI/XbaI and EcoRI/KpnI sites respectively without stop codon in pBAD24 and thereafter LdNLS was cloned at its C-terminus at XbaI/HindIII and KpnI/XbaI sites respectively. SDM constructs LdTOPIA^Y357A^ and LdTOPIA^E135A^ were generated by pfu-ultra DNA polymerase (Agilent technologies) using pBAD24-LdTOPIA as template. For functional complementation in antiTOPIA (transfectant parasite), the LdTOPIA, LdTOPIAΔNLS, LdTOPIL-*fus*-S (26) and LdRNaseH IIA (LdBPK_360700.1) were cloned in pXG vector at SmaI/BamHI sites. EcTOPIA and EcTOPIA + LdNLS cloned at SmaI site. All PCRs were done using Q5 DNA polymerase (NEB) and Restriction enzymes (NEB) for digestion. pXG was a kind gift from Prof. S. M. Beverley.

### Constructs for localization studies

All Constructs for transfection in *L. donovani* (DD8 strain) were cloned in pXG-GFP+2′ vector containing N-terminal GFP sequence and G418 resistance for selection in parasites. LdTOPIA and LdTOPIAΔNLS were cloned at BglII/NotI sites. For LdTOPIAΔNLS + SV40-NLS, LdTOPIAΔNLS was cloned without stop codon at BglII/NotI sites and SV40NLS was added at its C terminus by oligo annealing and ligation at NotI/BamHI sites. EcTOPIA was cloned at (BglII/NotI) and for EcTOPIA + LdNLS, pBAD24-EcTOPIA-LdNLS was used as template to PCR amplify the insert and cloned at BglII/NotI sites. The constructs express N-terminal GFP fusion proteins. LdTOPIA, LdTOPIAΔNLS, LdTOPIAΔNLS+SV40NLS, SV40NLS were cloned at XhoI/BamHI sites in EGFP mammalian expression vector containing N-terminal EGFP for localization studies in macrophages. For LdTOPIAΔNLS+SV40NLS, the insert was PCR amplified from pXG-GFP+2′-LdTOPIAΔNLS+SV40-NLS using XhoI forward primer and SV40NLS BamHI reverse primer and cloned in EGFP vector. SV40NLS was cloned at C-terminal to EGFP by oligo annealing. LdRNaseH IIA (LdBPK_360700.1) was cloned in pXG- ‘GFP+ at SmaI/BamHI sites. pXG-GFP +2’and pXG- ‘GFP+ was a kind gift from Prof. S. M. Beverley.

### Knockout by homologous recombination

For double knockout by homologous recombination, 400 bp flanking sequences 5′ and 3′ of *L. donovani* Topoisomerase IA gene from chromosome 21 were cloned in upstream and downstream of the HYG cassette and GFP cassette of pXG HYG and pXG GFP respectively. Four sets of primers were designed for cloning of the 400 bp flanks, both upstream (5′) and downstream (3′) of the two pXG plasmids mentioned above. The two flanks were to be cloned upstream and downstream of the HYG cassette (up-XhoI, down-SmaI) and GFP cassette (up-BamHI, down-EcoRI) in pXG HYG and pXG GFP respectively. pXG-GFP has two EcoRI sites, one at 2153 position downstream of GFP and another 6418 prior to the neomycin gene. To clone at the EcoRI site of pXG-GFP, the site prior to the neomycin gene was disrupted by site-directed mutagenesis. One base change was done, from GAATTC to GAGTTC. 30 μg of both the constructs prepared were digested with SalI restriction enzyme at 37 °C to linearize for electroporation. The plasmids were column purified using a plasmid purification kit. Plasmids were eluted using an electroporation buffer in 30 μl volume for each and electroporated in DD8 cells. The transfectants were selected by 100 μg/ml hygromycin and 100 μg/ml G418.

### Conditional mRNA antisense-mediated knockdown

For mRNA antisense in LtT7TR tetracycline dependent conditional expression strain, the 250 nt 5′UTR of LtTOPIA was cloned as the antisense construct in the HindIII/BamHI site of *pLew100v5-bleo* to express the antisense strand using primers 5′CGGGATCCCATACACATAACGAGCATAAAAACAG 3′ and 5′CCCAAGCTTCGCTGCTGTGCAGAAGAAATGACG 3′. It was electroporated (Experimental Procedures) into LtT7TR strain (21, 42) and transfectant was selected using bleomycin (100 μg/ml) and termed *antiTOPIA.* Empty vector transfectant were also prepared and termed *pL100*. Transcription was induced using tetracycline (1 μg/ml). pLew100v5 was a gift from George Cross (Addgene plasmid # 24011; http://n2t.net/addgene: 24011; RRID: Addgene_24011), (LtTOPIA-811 aa, locus LtaPh_2101400 chromosome 21, genomic location 31333 to 33768, Genbank GET88247.1).

### Electroporation in *Leishmania*

All electroporation was carried out by the following protocol. Exponential phase cells (1 × 10^8^) were washed twice with ice-cold 1× HBS (HEPES buffered saline). Cells were then washed once with electroporation buffer (21 mM HEPES pH 7.2, 137 mM NaCl, 5 mM KCl, 0.7 mM NaH_2_PO_4_, and 6 mM glucose) (25). Next, cells were counted and 4 × 10^7^ cells along with 10 to 30 μg of DNA were resuspended in an electroporation buffer and added in a 0.2 mm cuvette (Sigma). Next electroporation was carried out in a Bio-Rad gene pulser at 550 μF and 500V, one pulse- 5msec. Upon electroporation cells were transferred to M199 media with 20% FBS and G418 was added after 18 h, serial dilution-mediated clonal transfectant population was generated.

### Homology modeling and docking

The protein sequence of LdTOPIA Gene ID LdBPK_210180.1 was used to search potential templates in the Expasy Swiss model. The LdTOPIA homology modeled structure was generated based on template MtTOPIA PDB ID: 5D5H. Molecular dynamic simulation was done by GROMACS. Validation of stereochemical property and residue geometry was done by the PROCHECK tool. The norclomipramine structure was prepared by ChemDraw Ultra software and energy minimized by Chem3D Bio. The energy-minimized structures of LdTOPIA and norclomipramine were used for molecular docking using Autodock 4.2. Knowledge-based grid generation method was used to set the grids (43). The homology model structure of LdTOPIA was aligned to *E. coli* TOPIA (PDB ID: 4RUL) in RCSB PDB.

### Purification and antibody production

LdTOPIA and its SDM mutants were purified by expression in (LtT7TR) *Leishmania tarentolae* conditional expression system (20, 21). There is a HindIII restriction site at position 1915 bps of LdTOPIA. Hence an MCS was inserted in cloning sites, HindIII and BamHI of plew100v5-Bleo plasmid (Addgene). MCS designed HindIII/XhoI/NdeI/BamHI. Full length his tagged, 7x-HisLdTOPIA-6xHis were cloned in the MCS containing pLew100v5-bleo plasmid at XhoI/BamHI sites using forward primer 5′CCGCTCGAGATGCATCATCATCATCATCATCACATGTTGCGCCGCAGCGTGCGTGCG 3′ and reverse primer 5′ CGGGATCCCTAATGATGATGATGATGGTGCTTCTTCGGCTTCTTCGG 3′. The SDM and deletion mutants, LdTOPIA-Y357A, LdTOPIA-E135A, LdTOPIAΔNLS, LdTOPIA^1-684^ and LdTOPIA^1-640^ were generated using the pLew100v5-7xHis-LdTOPIA-6xHis construct as template. The constructs were transfected in LtT7TR and selected by bleomycin. Each time expression of 5 × 10^9^ cells was induced by 1 μg/ml tetracycline (Sigma). The induced cells were lysed in lysis buffer (50 mM Tris-Cl pH 7.5, 1 mM PMSF, 150 mM NaCl, 1 mM DTT, leupeptin, pepstatin) by sonication in ice using probe sonicator and centrifuged at high speed in cold. The supernatant containing overexpressed protein were passed through the equilibrated Ni-NTA (QIAGEN) column for binding. The column was subsequently washed with increasing concentration of imidazole. The proteins were eluted using 150 mM imidazole containing Tris-Cl buffer pH 7.5, 1 mM PMSF. The proteins were passed through S-200 column (Cytiva, size exclusion chromatography column) and peak fractions were pooled using Amicon Ultra Centrifugal Filters 10 kDa (Merck) in (50 mM Tris-Cl pH 7.5, 1 mM PMSF, 10% glycerol) and stored at −80 in aliquotes. The concentrations of proteins were measured using BSA standard curve. EcTOPIA gene was PCR amplified from *E. coli* genomic DNA and cloned in pET28a at NdeI/HindIII site of pET28a bacterial expression plasmid using Forward primer 5′-GGAATTCCATATGGGTAAAGCTCTTGTCATCG-3′ and reverse primer 5′-CCCAAGCTTTTATTTTTTTCCTTCAACCC-3′. It was overexpressed by 0.5 mM IPTG in BL21 DE3 strain at 22 °C for 12 h and purified by Ni-NTA chromatography as explained above. Catalytically inactive human RNaseH I (D210N) (24) cloned in pET15b at NdeI/BamHI sites were used to express with 0.5 mM IPTG at 16 °C for 12 h and purify from Rossetta DE3 pLysS strain by Ni-NTA and S-75 size exclusion chromatography as described above. Amicon Ultra Centrifugal Filters 3 kDa (Merck) used for buffer exchange. For antibody production LdTOPIA and GFP were purified from pET28a-LdTOPIA transformed Rosetta DE3 pLysS strain by 2M urea denaturation and pET28a-GFP Rosetta DE3 pLysS respectively. Rabbit anti-LdTOPIA and Rabbit anti-GFP antibodies were custom generated using the protein by Biobharati Pvt Ltd, India. Antibody dilutions for Western blot and immunofluorescence were standardized based on the guidelines in the datasheet.

### Cellular fractionation of *Leishmania* parasites

Cells were suspended in hypotonic buffer (10 mM Tris-HCl pH 7.5, 1 mM EDTA, 0.1 mM EGTA, 1 mM PMSF, Protease Inhibitor Cocktail, 5 mM DTT) and homogenized by a homogenizer, followed by centrifugation at 10,000 rpm for 20 min. The supernatant is a cytoplasmic fraction. The pellet was washed with the same buffer as before and centrifuged as above. The pellet was resuspended in high salt-containing buffer (400 mM NaCl, 1 mM EDTA, 20 mM Tris pH-7.5) and treated with DNase I and 6 mM MgCl_2_ for 30 min. The reaction was stopped by 6 mM EDTA and ultracentrifuged at 1, 65,000*g* at 4 °C for 1 h. The supernatant was used as a source of nuclear extract. Total cell lysates were prepared by incubating the cells in lysis buffer (NP-40 lysis buffer (CSH protocol), 1 mM PMSF, 1 mM DTT, 1× protease inhibitor cocktail, 1 mM EGTA and 1 mM EDTA) for 30 min in ice and centrifuged at 12,000 rpm, 20 min to separate the soluble and insoluble fraction. The Supernatant collected was total cell lysate.

### Immunoblotting

Cellular fractions from parasites were prepared as described earlier and equal amount of quantitated protein lysate was electrophoresed in 10% SDS-PAGE and, transferred to PVDF membrane (Merck) by semi-dry transfer blot system (Bio-Rad). Blocking was done using 5% skimmed milk and incubated with respective primary antibodies Rabbit anti-LdTOPIA antibody (custom-made, Biobharati Pvt Ltd), Rabbit anti-GFP antibody (custom made, Biobharati private limited), rabbit anti-Histone H3(CST) and Mouse anti-GAPDH (CST). Rabbit or Mouse secondary antibodies conjugated with HRP (Southern Biotech) were used for chemiluminescence detection using west pico luminol substrate (Thermo Scientific).

### DNA relaxation assay

DNA relaxation assay was carried out using negatively supercoiled pBluescript plasmid and purified LdTOPIA, LdTOPIA^-Y357A^, LdTOPIA^-E135A^, LdTOPIAΔNLS, LdTOPIA^-1-684^, LdTOPIA^-1-640^ in relaxation assay buffer (50 mM Tris-HCl, pH 7.5, 0.5 mM DTT, 100 mM NaCl, 10 mM MgCl_2_ and 50 μg/ml BSA, 0.5 mM EDTA, 5% glycerol) (25). Magnesium and salt dependency assays were carried out with assay buffers not containing MgCl_2_ and NaCl respectively. DNA and protein were incubated at various time points at 37 °C in a thermostat and electrophoresed in 1% TAE agarose gel without EtBr in 1× TAE running buffer and later visualized using 1 μg/ml EtBr staining. Inhibition assays were performed using drugs, 10, 20 and 40 μM of Imipramine (TCI) and Norclomipramine (Cayman Chemicals), 20 μM and 50 μM of Camptothecin (TCI) and Etoposide (Sigma) with LdTOPIA, and 10 μM of camptothecin and etoposide with Human TOPIB (Sigma) and Human TOPII (Sigma).

### Bi-directional gel electrophoresis

To distinguish between negative and positive topoisomers the DNA relaxation products were run in Bi-directions. The DNA relaxation assay was performed using *E. coli* DNA gyrase (NEB), Human TOPII (Sigma), and LdTOPIA using DNA relaxation assay buffer with negative and positive supercoil DNA (pBluescript) at 37 °C. The assay mixtures were run in 1% TBE agarose gel at constant 25V, 6h in 1× TBE buffer followed by 90° rotation and run in the second direction at constant 80V, 2 h in 1× TBE buffer containing 4 μg/ml chloroquine (44). Positive supercoiled DNA was generated by incubating fully relaxed DNA with recombinant reverse gyrase.

### Electrophoretic mobility shift assay

Purified LdTopIA (5–200 nM) were incubated with γ-32P (BRIT India) labeled 100 nM single and double-stranded oligos in buffer (50 mM Tris pH-7.5, 0.5 mM DTT, 100 mM NaCl,0.5 mM EDTA and 5% glycerol) for 15 min at 25 °C. [Oligo 5′ TATTGGGCGCTCTTCCGCTTCCTCGCTCACTG 3′] (25, 45). Double strandDNA was prepared by annealing the oligos at equimolar concentrations (1:1). The reaction mixtures were electrophoresed in 6% non-denaturing 0.5× TBE-polyacrylamide gel at 100 V, 4 °C and detected by autoradiography in phosphoimager. Labeling of Oligo with γ-32P at 5′ terminus was done by T4 polynucleotide kinase (NEB) and free nucleotides were removed by purifying the oligos using Qiagen purification columns.

### Measurement of DNA binding affinity by fluorescence polarization

A (fluorescein derivative) 5′FAM tagged DNA oligo 5′ TATTGGGCGCTCTTCCGCTTCCTCGCTCACTG3′, 5′ FAM tagged RNA oligo 5′ UAUUGGGCGCUCUUCCGCUUCCUCGCUCACUG 3′ and the complementary DNA oligo were custom synthesized (IDT technologies). The dsDNA fluorescent oligo was generated by oligo annealing using sense and antisense oligos at equimolar ratio. The 30 nM of both ssDNA and dsDNA were incubated with increasing concentrations of LdTOPIA (2–200 nM) in buffer 50 mM Tris pH-7.5, 0.5 mM DTT, 100 mM NaCl, 0.5 mM EDTA and 5% glycerol for 15 min at 25 °C. The 30 nM DNA: RNA duplex was incubated with catalytically inactive human RNaseH I (2–200 nM) for 15 min at 25 °C (24). The measurements were done using excitation wavelength 495 nm and emission wavelength 520 nm in Cytation 5 multimode reader.

(1) $$\text{Anisotropy},A=\left(I_{∥}-I_{⊥}\right)/\left(I_{∥}-2I_{⊥}\right)$$

The anisotropy values were fitted to the quadratic equation (2)

(2) $$\Delta A=\Delta A_{T}/2D_{T}\left\{\left(E_{T}+D_{T}+K_{D}\right)-{\left[{\left(E_{T}+D_{T}+K_{D}\right)}^{2}-4E_{T}D_{T}\right]}^{1/2}\right\}$$

where ΔA (A-A_0_) is the change in anisotropy, ΔA_T_ (A_max_-A_0_) is the total anisotropy change, E_T_ is the total enzyme concentration, D_T_ is the total DNA concentration, and K_D_ is the dissociation constant (25, 46). K_D_ value calculated using the formula (2). A_0_ and A_max_ are anisotropy for free and bound_max_ DNA. Fraction of bound DNA f_b_ was calculated by ΔA/ΔA_T_. The graph is the mean of three experiments. The K_D_ values calculated by Origin 8.5 software.

### Immunofluorescence analysis and imaging

The following protocol was carried out for immunofluorescence. *Leishmania* cells were washed twice with 1× PBS (phosphate buffer saline) and seeded in poly-lysine coated 10 well chamber slides or coverslips for 1 h to properly adhere cells at room temperature. The unadhered cells were washed with 1× PBS 4 to 5 times and fixed with 4% paraformaldehyde (Sigma) for 30 s and again washed with 1× PBS 4 to 5 times. The fixed cells were treated with 0.5% Triton X 100 (Sigma) for 15 min and subsequently washed, and incubated with 5% BSA (Sigma)in 1× PBS (blocking solution) for 1 h at room temperature. The blocking solution was removed and cells were incubated with the desired primary antibody for the incubation period as standardized for each antibody. This was followed by several washes and, incubation with required Alexa fluor 488 or Alexa 568 conjugated secondary antibodies (Invitrogen) for 1 h at room temperature. The cells were washed and stained with nuclear dye 1× DRAQ5 (CST) for 10 min. The cells were washed 6 to 7 times to remove excess stain followed by drying and mounting with anti-fade gold mounting medium (Invitrogen). For curing the slides were kept 24 h at room temperature and high-resolution imaging was done in a confocal laser scanning microscope (Olympus FV3000). DNA–RNA hybrid and dsDNA were detected by S9.6 antibody (Merck) and dsDNA antibody (Abcam), respectively.

### Complementation studies in *E. coli* RFM475 strain

*E. coli* RFM475 [TOPIA null mutant and GyrB ts (temperature sensitive)] strain was a kind gift from Prof. Marc Drolet (12). LdTOPIA, LdTOPIA deletion mutants, LdTOPIA active site SDM mutants, EcTOPIA, MtTOPIA, and LdTOPIL-*fus*-S were cloned in arabinose inducible pBAD24 plasmid as described earlier and transformed in RFM475 strain. Complementation studies were performed by growing cells in LB broth at non-permissive temperatures 30 °C and 42 °C in the presence of 0.2% arabinose. LB plates contained 100 μg/ml ampicillin and 0.2% arabinose. The transformed strains were at first grown at a permissive temperature of 37 °C in LB and these cultures were streaked in arabinose-containing LB plates for complementation studies.

### DNA: RNA hybrid immunoblotting (DRIB)

The following DRIB protocol was followed to detect R-loops (47, 48). The nucleus was isolated by hypotonic homogenization. Next, the nucleus was lysed by lysis buffer (1% SDS, 10 mM Tris-HCl pH 7.5, 1 mM EDTA, 100 mM NaCl, 50 μg/ml Proteinase K) at 55 °C for 2 h. This was followed by phenol: chloroform extraction and 20 μg/ml RNaseA (sigma) treatment for 30 min at 37 °C. The genomic DNA prepared was sonicated in Tris-HCl pH 7.5 with 100 mM NaCl buffer in a probe sonicator thrice for 30 s each in 20% amplitude to yield a maximum fragment size of around ∼1000 bps. For further nucleic acid fragmentation a cocktail of restriction enzymes and purified by phenol: chloroform extraction. This fragmented nucleic acid was transferred to Hybond N membrane (Merck) using a slot blot system and DNA: RNA hybrid was detected by S9.6 antibody (Merck). dsDNA was detected using anti-dsDNA antibody (abcam). Digestion of DNA: RNA hybrid was carried out using RNaseH (NEB).

### Modified MTT assay

Antisense TOPIA LtT7TR parasites were cultured and treated with 1 μg/ml tetracycline in RPMI1640 without phenol red media (Gibco) for 6 h, 12 h, 18 h, 24 h. Untreated and treated cells were then seeded to 96 well plate at density 5 × 10^5^ in 100 μl of same RPMI1640 to perform MTT assay. Since phenol red interfere in the absorbance, we chose this modified protocol of without phenol red media (49). The untreated and treated cells in 96 well plate were incubated with at a final concentration of 0.5 mg/ml MTT (TCI) and incubated for 3 h at 23 °C to form purple formazan. DMSO was used as solubilization solution to lyse cells, incubated for 10 min. Absorbance was measured at 590 nm in multimode reader (Cytation 5, BioTek). Graph plotted for percentage viability of cells over 24 h. To observe anti-leishmania property of TCAs, the *L. donovani* DD8 promastigotes and axenic amastigotes were treated with imipramine and norclomipramine at concentrations 2 to 100 μM for 72 h in RPMI 1640 and, similarly, BHU575 and BHU814 were treated with 2 to 100 μM norclomipramine. Modified MTT assay carried out as described earlier. The cytotoxic effect of norclomipramine on macrophages was observed by treating cells with 2 to 100 μM of the drug for 72 h in DMEM with phenol red media in 96-well plates. Thereafter, MTT at a final concentration of 0.5 mg/ml was added and incubated for 3 h at 37 °C. The media was removed from the wells and DMSO was used to solubilize cells. Absorbance is measured as described earlier. Subsequently, the graphs were plotted and IC50 values were calculated.

### Macrophage infection

RAW264.7 macrophage cells were infected with stationary phase *L. donovani* DD8-GFP promastigotes at 1: 10 ratio in DMEM media with 2% FBS. After 2 h incubation the cells were washed with 1× PBS to remove non-internalized parasites and infected cells were sorted based on green fluorescence of GFP in CytoFLEX SRT (Beckman Coulter). The sorted cells were seeded in 6-well plates for an additional 24 h with fresh complete media to convert promastigotes to amastigotes and thereafter treated with 10 μM NCL for 6 h, 24 h, and 48 h, and microscopic images were taken. The percentage of infected macrophages was obtained by flow cytometry analysis.

### Intracellular amastigote burden

The RAW264.7 macrophage cells were infected with DD8, BHU575, and BHU814 at 1: 10 ratio for 2 h, and upon removal of non-internalized parasites, the infected cells were kept in fresh complete media for the next 24 h as described earlier. Thereafter the infected cells were treated with 10 μM NCL for 48 h, and stained with Giemsa stain for 15 min upon methanol fixation. The number of amastigotes was calculated from macrophages by visualizing in a white field microscope and intracellular parasite burden was calculated as the number of *Leishmania* Units (LU) for both infected control and treated macrophages.

### Cell cycle analysis

Antisense TOPIA LtT7TR transfectant parasites at ∼5 × 10^5^ per ml cell density were treated with 1 μg/ml tetracycline for 6 h, 12 h, 18 h and 24 h in M199 media, 10% FBS. Next, the tetracycline untreated and treated parasites were centrifuged and washed twice with 1× phosphate buffer saline, pH-7.2 and finally resuspended at 10^6^ per ml cell density. The cells were stained with cell-permeable DNA binding dye DRAQ5 (CST) at 5 μM final concentration for 10 min at room temperature in FACS tube. Thereafter flow cytometry was carried out in FACS machine (Beckmen coulter) to obtain the percentage of cells in G_0_-G_1_, S, and G_2_-M phases of cell cycle.

### Statistical analysis

Results were analyzed with one-way ANOVA and Tukey’s post hoc test. *p* ≤ 0.05 was statistically significant. Error bars were represented as mean ± SD. Each experiment was independently performed at least three times. Graph plotted using Origin8.5. Technical replicates are mentioned as “n” where three independent biological replicate experiments have been carried out.

## Data availability

All data are included within manuscript. For additional information if any please contact somdeb@iitkgp.ac.in.

## supporting information

This article contains supporting information.

## Conflict of interest

The authors declare that they have no known competing financial interests or personal relationships that could have appeared to influence the work reported in this paper.

## References

[1] Martínez-Calvillo S., Vizuet-de-Rueda J.C., Florencio-Martínez L.E., Manning-Cela R.G., Figueroa-Angulo E.E. (2010). Gene expression in trypanosomatid parasites. J. Biomed. Biotechnol..

[2] Pita S., Díaz-Viraqué F., Iraola G., Robello C. (2019). The Tritryps comparative repeatome: insights on repetitive element evolution in trypanosomatid pathogens. Genome Biol. Evol..

[3] Lye L.F., Owens K., Shi H., Murta S.M.F., Vieira A.C., Turco S.J. (2010). Retention and loss of RNA interference pathways in trypanosomatid protozoans. PLoS Pathog..

[4] Dasgupta T., Ferdous S., Tse-Dinh Y.C. (2020). Mechanism of type IA topoisomerases. Molecules.

[5] Champoux J.J. (2001). DNA topoisomerases: structure, function, and mechanism. Annu. Rev. Biochem..

[6] Scocca J.R., Shapiro T.A. (2008). A mitochondrial topoisomerase IA essential for late theta structure resolution in African trypanosomes. Mol. Microbiol..

[7] Dorman C.J. (2019). DNA supercoiling and transcription in bacteria: a two-way street. BMC Mol. Cell Biol..

[8] Massé E., Drolet M. (1999). Escherichia coli DNA topoisomerase I inhibits R-loop formation by relaxing transcription-induced negative supercoiling. J. Biol. Chem..

[9] Gowrishankar J., Leela J.K., Anupama K. (2013). R-loops in bacterial transcription: their causes and consequences. Transcription.

[10] Windgassen T.A., Wessel S.R., Bhattacharyya B., Keck J.L. (2018). Mechanisms of bacterial DNA replication restart. Nucleic Acids Res..

[11] Belotserkovskii B.P., Tornaletti S., D'Souza A.D., Hanawalt P.C. (2018). R-loop generation during transcription: formation, processing and cellular outcomes. DNA Repair (Amst).

[12] Cristini A., Ricci G., Britton S., Salimbeni S., Huang S.Y.N., Marinello J. (2019). Dual processing of R-loops and topoisomerase I induces transcription-dependent DNA double-strand breaks. Cell Rep..

[13] Pan X., Jiang N., Chen X., Zhou X., Ding L., Duan F. (2014). R-loop structure: the formation and the effects on genomic stability. Yi Chuan.

[14] Drolet M., Phoenix P., Menzel R., Massé E., Liu L.F., Crouch R.J. (1995). Overexpression of RNase H partially complements the growth defect of an Escherichia coli delta topA mutant: R-loop formation is a major problem in the absence of DNA topoisomerase I. Proc. Natl. Acad. Sci. U. S. A..

[15] Costa-Silva H.M., Resende B.C., Umaki A.C.S., Prado W., da Silva M.S., Virgílio S. (2021). DNA topoisomerase 3α is involved in homologous recombination repair and replication stress response in *Trypanosoma cruzi*. Front. Cell Dev. Biol..

[16] Pommier Y., Nussenzweig A., Takeda S., Austin C. (2022). Human topoisomerases and their roles in genome stability and organization. Nat. Rev. Mol. Cell Biol..

[17] Brugiolo M., Herzel L., Neugebauer K.M. (2013). Counting on co-transcriptional splicing. F1000prime Rep..

[18] Niu D.K. (2007). Protecting exons from deleterious R-loops: a potential advantage of having introns. Biol. Direct..

[19] Bonnet A., Grosso A.R., Elkaoutari A., Coleno E., Presle A., Sridhara S.C. (2017). Introns protect eukaryotic genomes from transcription-associated genetic instability. Mol. Cell.

[20] Klatt S., Hartl D., Fauler B., Gagoski D., Castro-Obregón S., Konthur Z. (2013). Generation and characterization of a Leishmania tarentolae strain for site-directed *in vivo* biotinylation of recombinant proteins. J. Proteome Res..

[21] Basile G., Peticca M. (2009). Recombinant protein expression in Leishmania tarentolae. Mol. Biotechnol..

[22] Wight M., Werner A. (2013). The functions of natural antisense transcripts. Essays Biochem..

[23] Achenbach T.V., Brunner B., Heermeier K. (2003). Oligonucleotide-based knockdown technologies: antisense versus RNA interference. Chembiochem.

[24] Chen L., Chen J.Y., Zhang X., Gu Y., Xiao R., Shao C. (2017). R-ChIP using inactive RNase H reveals dynamic coupling of R-loops with transcriptional pausing at gene promoters. Mol. Cell.

[25] BoseDasgupta S., Ganguly A., Das B.B., Roy A., Khalkho N.V., Majumder H.K. (2008). The large subunit of Leishmania topoisomerase I functions as the 'molecular steer' in type IB topoisomerase. Mol. Microbiol..

[26] Das B.B., Bose Dasgupta S., Ganguly A., Mazumder S., Roy A., Majumder H.K. (2007). Leishmania donovani bisubunit topoisomerase I gene fusion leads to an active enzyme with conserved type IB enzyme function. FEBS J..

[27] Briggs E., Crouch K., Lemgruber L., Hamilton G., Lapsley C., McCulloch R. (2019). Trypanosoma brucei ribonuclease H2A is an essential R-loop processing enzyme whose loss causes DNA damage during transcription initiation and antigenic variation. Nucleic Acids Res..

[28] Godbole A.A., Ahmed W., Bhat R.S., Bradley E.K., Ekins S., Nagaraja V. (2015). Targeting Mycobacterium tuberculosis topoisomerase I by small-molecule inhibitors. Antimicrob. Agents Chemother..

[29] Tan K., Cao N., Cheng B., Joachimiak A., Tse-Dinh Y.C. (2016). Insights from the structure of Mycobacterium tuberculosis topoisomerase I with a novel protein fold. J. Mol. Biol..

[30] Mukherjee S., Mukherjee B., Mukhopadhyay R., Naskar K., Sundar S., Dujardin J.C. (2012). Imipramine is an orally active drug against both antimony sensitive and resistant Leishmania donovani clinical isolates in experimental infection. PLoS Negl. Trop. Dis..

[31] Mukherjee S., Mukherjee B., Mukhopadhyay R., Naskar K., Sundar S., Dujardin J.C., Roy S. (2014). Imipramine exploits histone deacetylase 11 to increase the IL-12/IL-10 ratio in macrophages infected with antimony-resistant Leishmania donovani and clears organ parasites in experimental infection. J. Immunol..

[32] Manzo S.G., Hartono S.R., Sanz L.A., Marinello J., De Biasi S., Cossarizza A. (2018). DNA Topoisomerase I differentially modulates R-loops across the human genome. Genome Biol..

[33] Cristini A., Tellier M., Constantinescu F., Accalai C., Albulescu L.O., Heiringhoff R. (2022). RNase H2, mutated in Aicardi-Goutières syndrome, resolves co-transcriptional R-loops to prevent DNA breaks and inflammation. Nat. Commun..

[34] Amon J.D., Koshland D. (2016). RNase H enables efficient repair of R-loop induced DNA damage. Elife.

[35] Kevric I., Cappel M.A., Keeling J.H. (2015). New world and old world leishmania infections: a practical review. Dermatol. Clin..

[36] Kasozi K.I., Zirintunda G., Ssempijja F., Buyinza B., Alzahrani K.J., Matama K. (2021). Epidemiology of trypanosomiasis in wildlife-implications for humans at the wildlife interface in africa. Front. Vet. Sci..

[37] da Silva Rodrigues J.H., Miranda N., Volpato H., Ueda-Nakamura T., Nakamura C.V. (2019). The antidepressant clomipramine induces programmed cell death in Leishmania amazonensis through a mitochondrial pathway. Parasitol. Res..

[38] Pandey R.K., Verma P., Sharma D., Bhatt T.K., Sundar S., Prajapati V.K. (2016). High-throughput virtual screening and quantum mechanics approach to develop imipramine analogues as leads against trypanothione reductase of leishmania. Biomed. Pharmacother..

[39] Burza S., Sinha P.K., Mahajan R., Sanz M.G., Lima M.A., Mitra G. (2014). Post Kala-Azar dermal leishmaniasis following treatment with 20 mg/kg liposomal amphotericin B (Ambisome) for primary visceral leishmaniasis in Bihar, India. PLoS Negl. Trop. Dis..

[40] Zijlstra E.E., Alves F., Rijal S., Arana B., Alvar J. (2017). Post-kala-azar dermal leishmaniasis in the Indian subcontinent: a threat to the South-East Asia region kala-azar elimination programme. PLoS Negl. Trop. Dis..

[41] Ponte-Sucre A., Gamarro F., Dujardin J.C., Barrett M.P., López-Vélez R., García-Hernández R. (2017). Drug resistance and treatment failure in leishmaniasis: a 21st century challenge. PLoS Negl. Trop. Dis..

[42] Bandini G., Damerow S., Sempaio Guther M.L., Guo H., Mehlert A., Paredes Franco J.C. (2021). An essential, kinetoplastid-specific GDP-Fuc: β-D-Gal α-1,2-fucosyltransferase is located in the mitochondrion of *Trypanosoma brucei*. Elife.

[43] Bhattacharya S., Junghare V., Pandey N.K., Ghosh D., Patra H., Hazra S. (2020 Feb 15). An insight into the complete biophysical and biochemical characterization of novel class A beta-lactamase (Bla1) from Bacillus anthracis. Int. J. Biol. Macromol..

[44] Gibson E.G., Oviatt A.A., Osheroff N. (2020). Two-dimensional gel electrophoresis to resolve DNA topoisomers. Methods Mol. Biol..

[45] Lee D.G., Makhov A.M., Klemm R.D., Griffith J.D., Bell S.P. (2000). Regulation of origin recognition complex conformation and ATPase activity: differential effects of single-stranded and double-stranded DNA binding. EMBO J..

[46] Valuchova S., Fulnecek J., Petrov A.P., Tripsianes K., Riha K. (2016). A rapid method for detecting protein-nucleic acid interactions by protein induced fluorescence enhancement. Sci. Rep..

[47] Gibbons H.R., Aune T.M. (2020). Immunoprecipitation of DNA: RNA hybrids using the S9.6 antibody. Methods Mol. Biol..

[48] Crossley M.P., Bocek M.J., Hamperl S., Swigut T., Cimprich K.A. (2020). qDRIP: a method to quantitatively assess RNA-DNA hybrid formation genome-wide. Nucleic Acids Res..

[49] Dutta A., Bandyopadhyay S., Mandal C., Chatterjee M. (2005). Development of a modified MTT assay for screening antimonial resistant field isolates of Indian visceral leishmaniasis. Parasitol. Int..

