# Supplementary material for: Resolving the polycistronic aftermath: Essential role of topoisomerase IA in preventing R-loops in Leishmania
Source: J Biol Chem. 2024 Mar 12;300(4):107162. doi: 10.1016/j.jbc.2024.107162 (PMC11021369; doi:10.1016/j.jbc.2024.107162)
Supplement: Supplementary Table 1 [file mmc1.docx]

**Supplementary Table 1**

**List of oligonucleotides**

| **Clone** | **Primers Name**  **Forward (F)**  **Reverse (R)** | **Sequence (5’-3’)** |
| --- | --- | --- |
| pET28A-LdTOPIA | pET28A-TOPIANdeF | GGAATTCCATATGTTGCGCCGCAGCGTGCGTG |
|  | pET28A-TOPIAXhoR | CCGCTCGAGCTACTTCTTCGGCTTCTTCGGCTT |
| pLew100v5-7X-His-LdTOPIA-6X-His | 7X-HisLdTOPIAXhoF | CCGCTCGAGATGCATCATCATCATCATCATCACATGTTGCGCCGCAGCGTGCGTGCG |
|  | 6X-HisLdTOPIABamR | CGGGATCCCTAATGATGATGATGATGGTGCTTCTTCGGCTTCTTCGG |
| pET28A-EcTOPIA | EcTOPIANdeF | GGAATTCCATATGGGTAAAGCTCTTGTCATCGTT |
|  | EcTOPIAHindR | CCCAAGCTTTTATTTTTTTCCTTCAACCC |
| pBAD24-LdTOPIA | pBADHisEcoF | CGGAATTCATGCATCATCATCATCATCAC |
|  | pBADTOPIAXbR | GCTCTAGACTACTTCTTCGGCTTCTTCGGCTT |
| pBAD24-LdTOPIA^∆NLS^ | pET28A-TOPIANdeF | GGAATTCCATATGTTGCGCCGCAGCGTGCGTG |
|  | LdTOPIA∆NLSXbaR | GCTCTAGACTACTTTACGGAGCTGCTGCTGCTGCC |
| pBAD24-LdTOPIA1-771 | pET28A-TOPIANdeF | GGAATTCCATATGTTGCGCCGCAGCGTGCGTG |
|  | 771XbaR | GCTCTAGACTAGCGAGCGGTTCCCGAGGATG |
| pBAD24-LdTOPIA1-684 | pET28A-TOPIANdeF | GGAATTCCATATGTTGCGCCGCAGCGTGCGTG |
|  | TIA-684XbR | GCTCTAGA CTATCGGTGTGCCTTGGTCGC |
| pBAD24-LdTOPIA1-640 | pET28A-TOPIANdeF | GGAATTCCATATGTTGCGCCGCAGCGTGCGTG |
|  | 640XbaR | GCTCTAGACTAAAGCTTTTTCTCAAACGCGGAGGT |
| pBAD24-EcTOPIA with stop codon | EcTOPIANdeF | GGAATTCCATATGGGTAAAGCTCTTGTCATCGTT |
|  | EcTOPIAXbaR1 | GCTCTAGATTATTTTTTTCCTTCAACCC |
| pBAD24-EcTOPIA without stop codon | EcTOPIANdeF | GGAATTCCATATGGGTAAAGCTCTTGTCATCGTT |
|  | EcTOPIAXbaR2 | GCTCTAGATTTTTTTCCTTCAACCC |
| pBAD24-EcTOPIA without stop codon +LdNLS | TOPIA-NLSXbaF | GCTCTAGAAAAAAGGAGTCACACGGGGC |
|  | TOPIA-NLSHindR | CCCAAGCTTCTACTTCTTCGGCTTCTTCGGCTT |
| pBAD24-MtTOPIA | MtTOPEcoRF | CGGAATTCATGTTGGCTGACCCGAAAACGAAGGGC |
|  | MtTOPKpnIR | CGGGGTACCCTAGTCGCGCTTGGCTGCCTTCTT |
| pBAD24-MtTOPIA- without stop codon +LdNLS | MtTOPEcoRF | CGGAATTCATGTTGGCTGACCCGAAAACGAAGGGC |
|  | MtTOPKpnIR2 | CGGGGTACCGTCGCGCTTGGCTGCCTTCTT |
|  | TOPIA-NLSKpnIF | CGGGGTACCAAAAAGGAGTCACACGGGGC |
|  | pBADTOPIAXbR | GCTCTAGA CTACTTCTTCGGCTTCTTCGGCTT |
| pBAD24-LdTOPL-*fus*-S | LdTOPL-*fus*-SNdeIF | GGAATTCCATATGAAGGTGGAGAATAGC |
|  | LdTOPL-*fus*-SXbaIR | GCTCTAGATCAAAAATCGAAGTTCTCGGC |
| pXG-GFP+2’-LdTOPIA | LdTOPIABglF | GAAGATCTATGTTGCGCCGCAGCGTGCGTGCG |
|  | LdTOPIANotR | AAGGAAAAAAGCGGCCGCCTACTTCTTCGGCTTCTTCGGCTT |
| pXG-GFP+2’-LdTOPIA-ΔNLS | LdTOPIABglF | GAAGATCTATGTTGCGCCGCAGCGTGCGTGCG |
|  | LdTopdelNlNotR1 | AAGGAAAAAAGCGGCCGCCTACTTTACGGAGCTGCTGCTGCTGCC |
| pXG-GFP+2’-LdTOPIA-ΔNLS 2 | LdTOPIABglF | GAAGATCTATGTTGCGCCGCAGCGTGCGTGCG |
|  | LdTopdelNlNotR2 | AAGGAAAAAAGCGGCCGCCTTTACGGAGCTGCTGCTGCTGCC |
| pXG-GFP+2’-LdTOPIA-ΔNLS 2+ SV40NLS | SV40NotF-oligo | GGCCGCCCGAAGAAGAAGAGAAAGGTGTAGG |
|  | SV40BamR-oligo | GATCCCTACACCTTTCTCTTCTTCTTCGGGC |
| pXG-GFP+2’-EcTOPIA | EcTOPBglF | GAAGATCTATGGGTAAAGCTCTTGTCATCGTT |
|  | EcTOPNotR | AAGGAAAAAAGCGGCCGCTTATTTTTTTCCTTCAACCCA |
| pXG-GFP+2’-EcTOPIA- LdNLS | EcTOPBglF | GAAGATCTATGGGTAAAGCTCTTGTCATCGTT |
|  | TOPIA-NLSNotR | AAGGAAAAAAGCGGCCGCCTACTTCTTCGGCTTCTTCGGC |
| EGFP-LdTOPIA | LdTOPIAXhoF | CCGCTCGAGATGTTGCGCCGCAGCGTGCGTGCG |
|  | LdTOPIABamHR | CGGGATCCCTACTTCTTCGGCTTCTTCGGCTT |
| EGFP-LdTOPIA∆NLS | LdTOPIAXhoF | CCGCTCGAGATGTTGCGCCGCAGCGTGCGTGCG |
|  | LdTOPIA∆NLSBamR | CGGGATCCCTACTTTACGGAGCTGCTGCTGCTGC |
| EGFP-LdTOPIA∆NLS+SV40NLS | LdTOPIAXhoF | CCGCTCGAGATGTTGCGCCGCAGCGTGCGTGCG |
|  | SV40NLSBamR | CGGGATCCCTACACCTTTCTCTTCTTCTTCGG |
| EGFP-SV40NLS | SV40XhoF-oligo | TCGAGCCGAAGAAGAAGAGAAAGGTGTAGG |
|  | SV40BamR-oligo | GATCCCTACACCTTTCTCTTCTTCTTCGGC |
| pBAD24-LdTOPIA^Y357A^ | T1AY357Af | CAGATGGGCTACATCACCGCCCCGCGCACGGACAGCACC |
|  | T1AY357Ar | GGTGCTGTCCGTGCGCGGGGCGGTGATGTAGCCCATCTG |
| pBAD24-LdTOPIA^E135A^ | T1AE135Af | GCGACCGATCCCGATCGTGCGGGAGAGCTGATCGCGGTG |
|  | T1AE135Ar | CACCGCGATCAGCTCTCCCGCACGATCGGGATCGGTCGC |
| pLew100v5-LtTOPIA 5’UTR | 5’UTRHindF | CCCAAGCTTCGCTGCTGTGCAGAAGAAATGACG |
|  | 5’UTRBamR | CGGGATCCCATACACATAACGAGCATAAAAACAG |
| pXG-‘GFP+ SDM | EcoRISDMF | GCTTGACGCATACGCGACGAGTTCGAAAGCTCACCTCATTCCTCCC |
|  | EcoRISDMR | GGGAGGAATGAGGTGAGCTTTCGAACTCGTCGCGTATGCGTCAAGC |
| 5’ flank in pXG HYG | 5’ pXG-HYGF | CCGCTCGAGGCACGAACCTTCTCTCCCCCTCCCT |
|  | 5’ pXG-HYGR | CCGCTCGAGTAGCTGCTGTGCAGAAGAAATGATG |
| 3’ Flank in pXG HYG | 3’ pXG-HYGF | TCCCCCGGGAAGAAGTAGCGCATGGGCCCACGCG |
|  | 3’ pXG-HYGR | TCCCCCGGGTAGCCGCCGGCTTGAGCGCACTGTA |
| 5’ flank in pXG-‘ GFP+ | 5’ pXG-GFPF | CGGGATCCGCACGAACCTTCTCTCCCCCTCCCT |
|  | 5’ pXG-GFPR | CGGGATCCTAGCTGCTGTGCAGAAGAAATGATG |
| 3’ flank in pXG-‘ GFP+ | 3’ pXG-GFPF | GGAATTCAAGAAGTAGCGCATGGGCCCACGCG |
|  | 3’ pXG-GFPR | GGAATTCTAGCCGCCGGCTTGAGCGCACTGTA |
| pXG-LdTOPIA | LdTOPIASmaF | TCCCCCGGGATGTTGCGCCGCAGCGTGCGTGCG |
|  | LdTOPIABamR | CGGGATCCCTACTTCTTCGGCTTCTTCGGCTT |
| pXG-LdTOPIA∆NLS | LdTOPIASmaF | TCCCCCGGGATGTTGCGCCGCAGCGTGCGTGCG |
|  | LdTOPIA∆NLSBamR | CGGGATCCCTACTTTACGGAGCTGCTGCTGCTGC |
| pXG-LdTOPIA∆NLS-SV40NLS | LdTOPIASmaF | TCCCCCGGGATGTTGCGCCGCAGCGTGCGTGCG |
|  | SV40NLSBamR | CGGGATCCCTACACCTTTCTCTTCTTCTTCGG |
| pXG-EcTOPIA | EcTOPSmaF | TCCCCCGGGATGGGTAAAGCTCTTGTCATCGTT |
|  | EcTOPSmaR | TCCCCCGGGTTATTTTTTTCCTTCAACCCA |
| pXG-EcTOPIA-LdNLS | EcTOPSmaF | TCCCCCGGGATGGGTAAAGCTCTTGTCATCGTT |
|  | TOPIA-NLSSmaR | TCCCCCGGGCTACTTCTTCGGCTTCTTCGGC |
| pLew100v5-LdTOPIA-dNLS | 7X-HisLdTOPIAXhoF | CCGCTCGAGATGCATCATCATCATCATCATCACATGTTGCGCCGCAGCGTGCGTGCG |
|  | LdTOPIA∆NLSBamR | CGGGATCCCTACTTTACGGAGCTGCTGCTGCTGC |
| pLew100v5-LdTOPIA-684 | 7X-HisLdTOPIAXhoF | CCGCTCGAGATGCATCATCATCATCATCATCACATGTTGCGCCGCAGCGTGCGTGCG |
|  | LdTOPIA 684BamR | CGGGATCCCTATCGGTGTGCCTTGGTCGC |
| pLew100v5-LdTOPIA-640 | 7X-HisLdTOPIAXhoF | CCGCTCGAGATGCATCATCATCATCATCATCACATGTTGCGCCGCAGCGTGCGTGCG |
|  | LdTOPIA-640BamR | CGGGATCCCTAAAGCTTTTTCTCAAACGCGGAGGT |
| pET15b-HsRNaseHI | HsRNaseHINdeIF | GGAATTCCATATGAGCTGGCTTCTGTTCCTGGC |
|  | HsRNaseHIBamHR | CGGGATCCTCAGTCTTCCGATTGTTTAGC |
| HsRNaseHI D210N SDM | Sense oligo | CTGGTTCTGTATACAAACAGTATGTTTACGATA |
|  | Antisense oligo | TATCGTAAACATACTGTTTGTATACAGAACCAG |
| pXG-LdTOPIL-fus-S | L-*fus*-S SmaIF | TCCCCCGGGATGAAGGTGGAGAATAGC |
|  | L-*fus*-S BamHR | CGGGATCCTCAAAAATCGAAGTTCTCGGC |
| pXG-LdRNaseHIIA | LdRNaseH IISmaIf | TCCCCCGGG ATGACGGATACGCCATCTACTCTC |
|  | LdRNaseH IIBamHR | CGGGATCCCTACTCCACCAAGCTGGTTGGGTA |
| pXG- ‘GFP+ LdRNaseHII | LdRNaseH IISmaIf | TCCCCCGGGATGACGGATACGCCATCTACTCTC |
|  | LdRNaseH IIBamHR | CGGGATCCCTCCACCAAGCTGGTTGGGTA |
