# Supplementary material for: Resolving the polycistronic aftermath: Essential role of topoisomerase IA in preventing R-loops in Leishmania
Source: J Biol Chem. 2024 Mar 12;300(4):107162. doi: 10.1016/j.jbc.2024.107162 (PMC11021369; doi:10.1016/j.jbc.2024.107162)
Supplement: Supplementary Figures 1–6 [file mmc2.docx]

**Supporting Information**

**Figure S1**


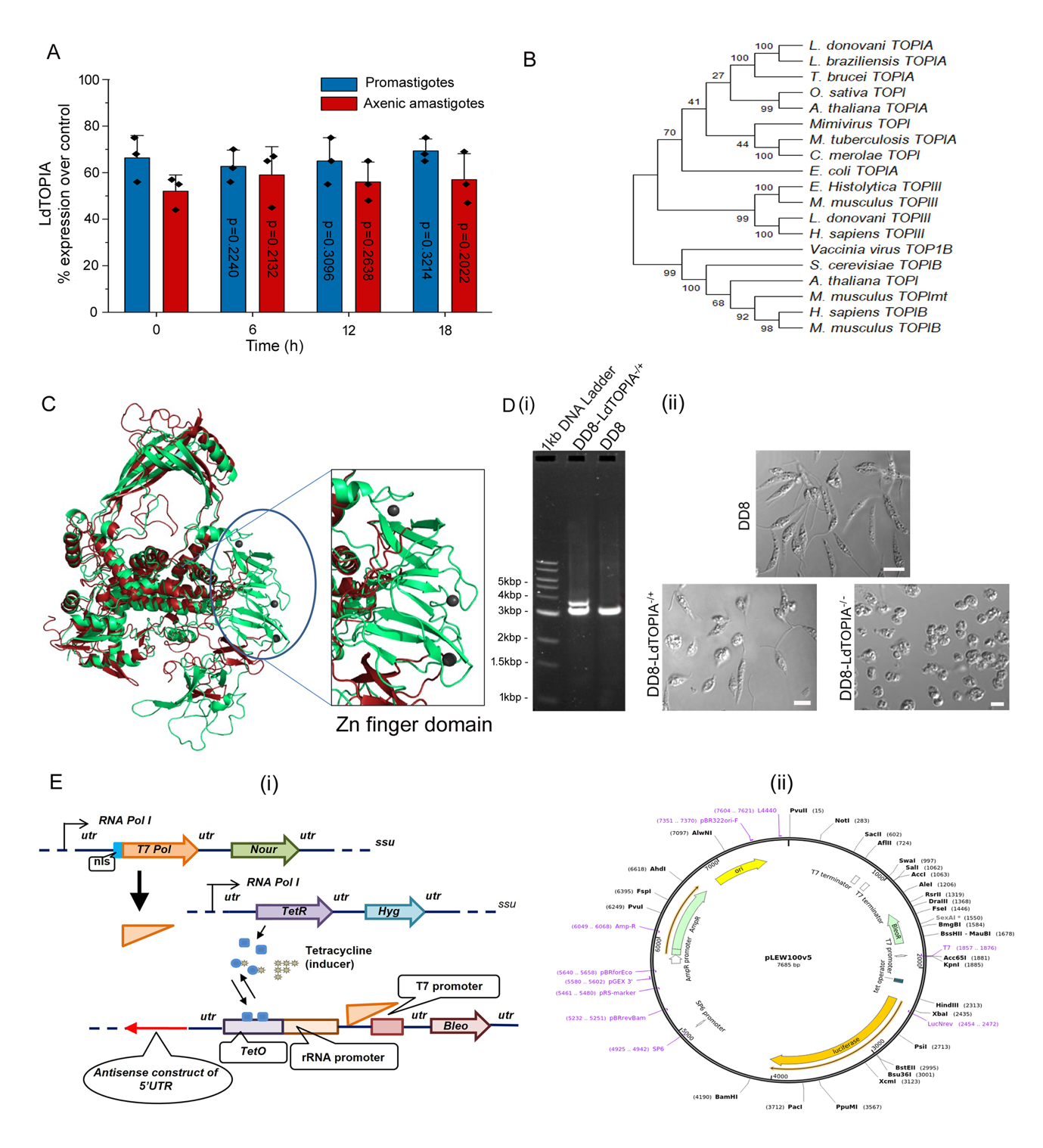


Figure S1: (A)Quantitation of LdTOPIA expression in *L. donovani* DD8 promastigotes and axenic amastigotes expressed as percentage over control. n=3, each dot represent mean of three biological replicates from each independent experiment, Bar represent mean ± SD. P vs. respective control (0h). (B)Phylogenetic analysis of Type I topoisomerases from various organisms obtained using MegaX11. (C) Homology modeled LdTOPIA structure (Brown) overlapped over EcTOPIA structure (PDB ID:4RUL) (Green) exhibiting the absence of Zn finger domain in LdTOPIA. (D) Homologous recombination mediated generation of LdTOPIA knockout parasites where (i) the pXG-HYG containing 400bps sequence of the 5’ and 3’ flanking region where one allele of LdTOPIA is replaced by hygromycin was observed and (ii) the slow growing stressed phenotype of the parasites observed under the microscope. Scale bar: 5μm (E) (i) Strategy for tetracycline inducible conditional antisense or expression system of *Leishmania tarentolae*. The T7 promoter and tetracycline repressor genes are genome integrated and selected using nourseothricin and hygromycin respectively to generate the *L. tarentolae* LtT7TR strain. For conditional expression of antisense mRNA, 250bp of the 5’UTR of LtTOPIA before the start codon was cloned in the (ii) pLew100v5-Bleo vector at HindIII/BamHI sites. Upon tetracycline addition its binding to Tet Repressor induces its structural change and therefore its failure to bind to Tet Operator. This sets free T7 RNA pol mediated transcription.

**Figure S2**


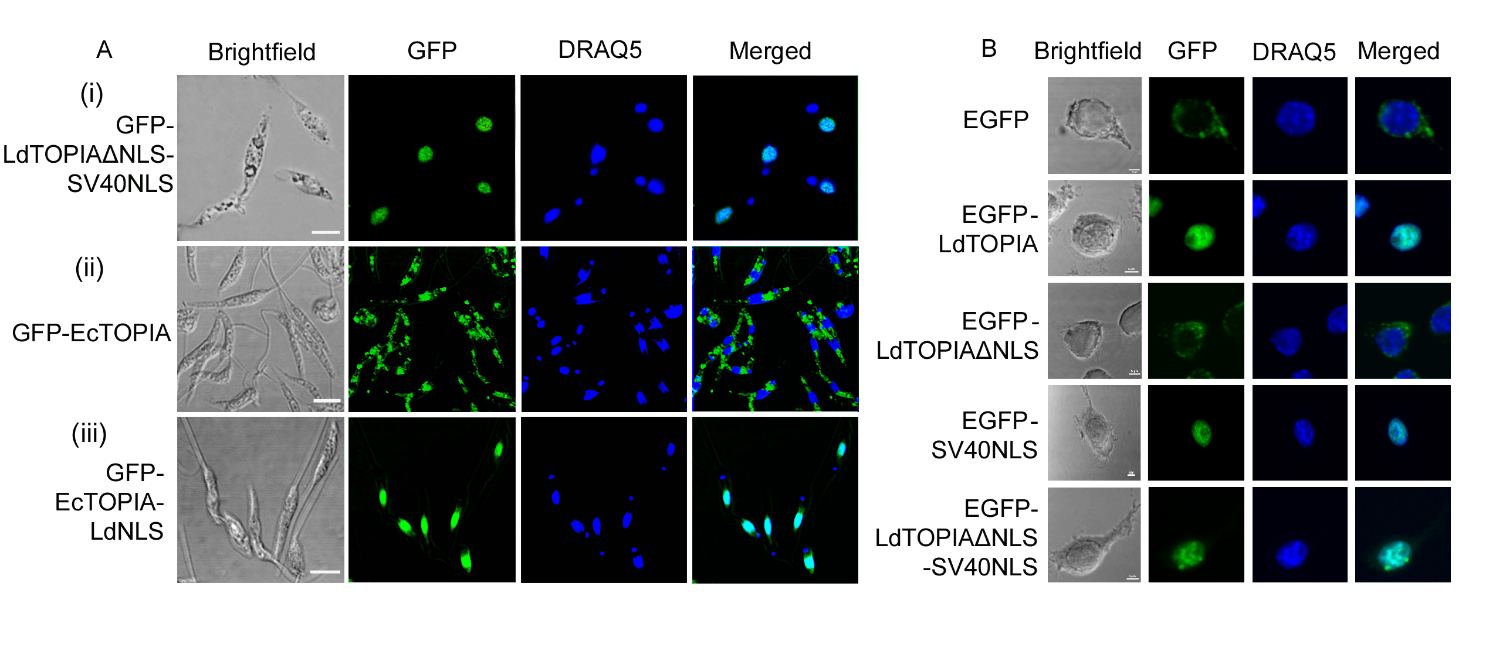


Figure S2:(A) Localization studies of LdTOPIA mutants and other constructs. Microscopic images of (i) NLS deleted LdTOPIA tagged with SV40 T-antigen NLS, ii) EcTOPIA and (iii) EcTOPIA tagged with LdNLS and cloned in pXG-GFP+2’, Scale bar, 5µm. (B) Heterologous expression and localization of EGFP-LdTOPIA, EGFP-LdTOPIAΔNLS, EGFP-SV40NLS and EGFP-LdTOPIAΔNLS-SV40NLS cloned in EGFP vector and transfected into RAW264.7 macrophages. All the constructs of *Leishmania* transfection were in pXG-GFP+2’ plasmid and for heterologous system (macrophages), EGFP constructs were prepared. Scale bar, 5µm.

**Figure S3**


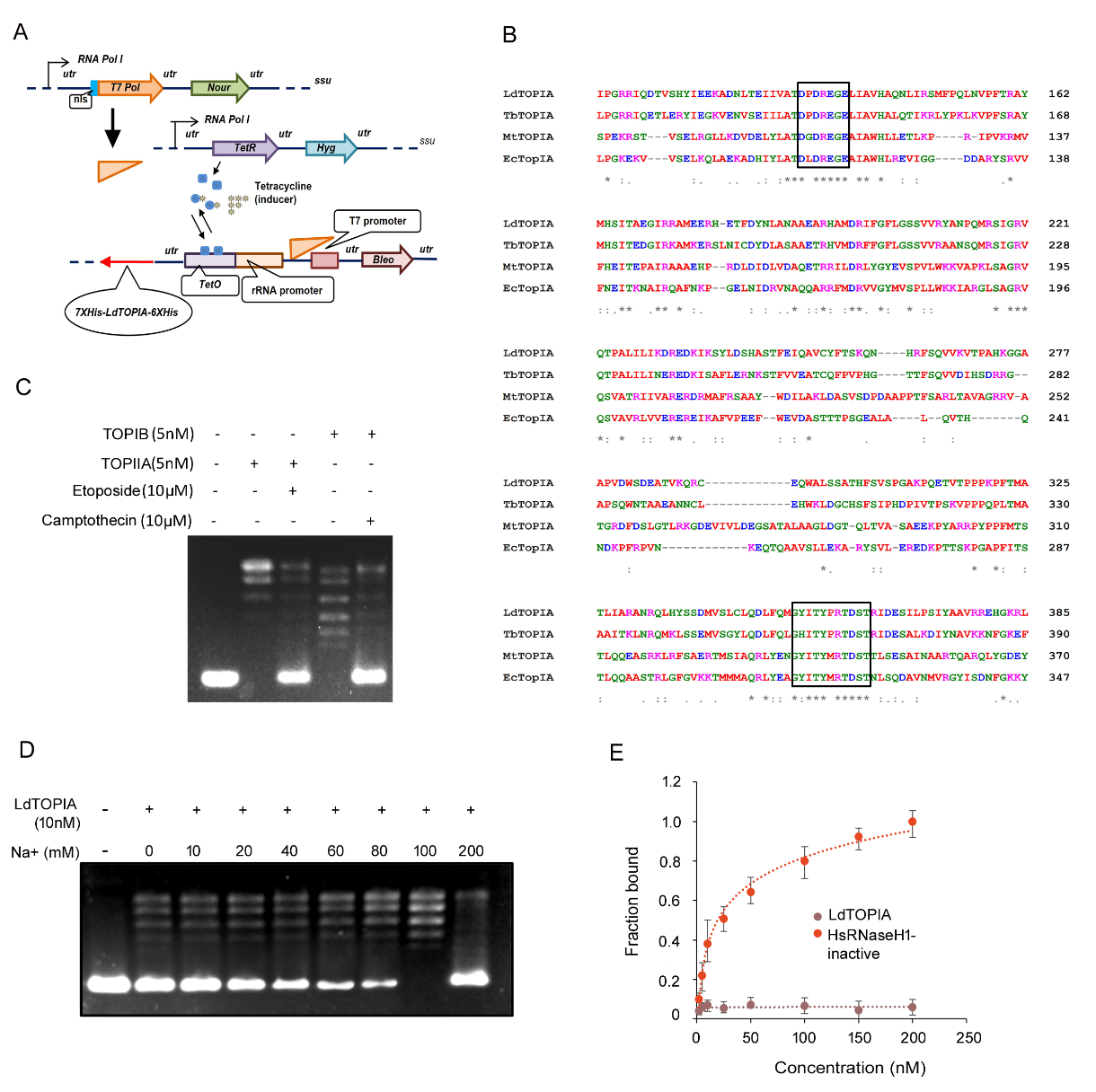


Figure S3: (A) Schematic representation of Tet inducible expression of LdTOPIA in LtT7TR strain. 7XHis-LdTOPIA-6XHis was cloned in pLew100v5 vector as described in material and methods. (B) Regions showing consensus sequence (DXDREGE) and conserved active site GYITYPRTDST by multiple sequence alignment. (C) Human TOPIB and TOPIIA incubated with (-) SC pBluescript DNA in absence and presence of camptothecin (CPT) and etoposide (Etop). (D) DNA relaxation using LdTOPIA and (-) SC pBluescript DNA in presence of increasing NaCl concentration as indicated. (E) DNA: RNA duplex binding affinity was measured by fluorescence polarization using 5’ FAM tagged substrate incubated with increasing concentration of LdTOPIA and catalytically inactive Human RNaseH1. Fraction bound values were plotted against enzyme concentrations (2-200nM). (n=5 and 3 biological replicate, mean ± SD)

**Figure S4**


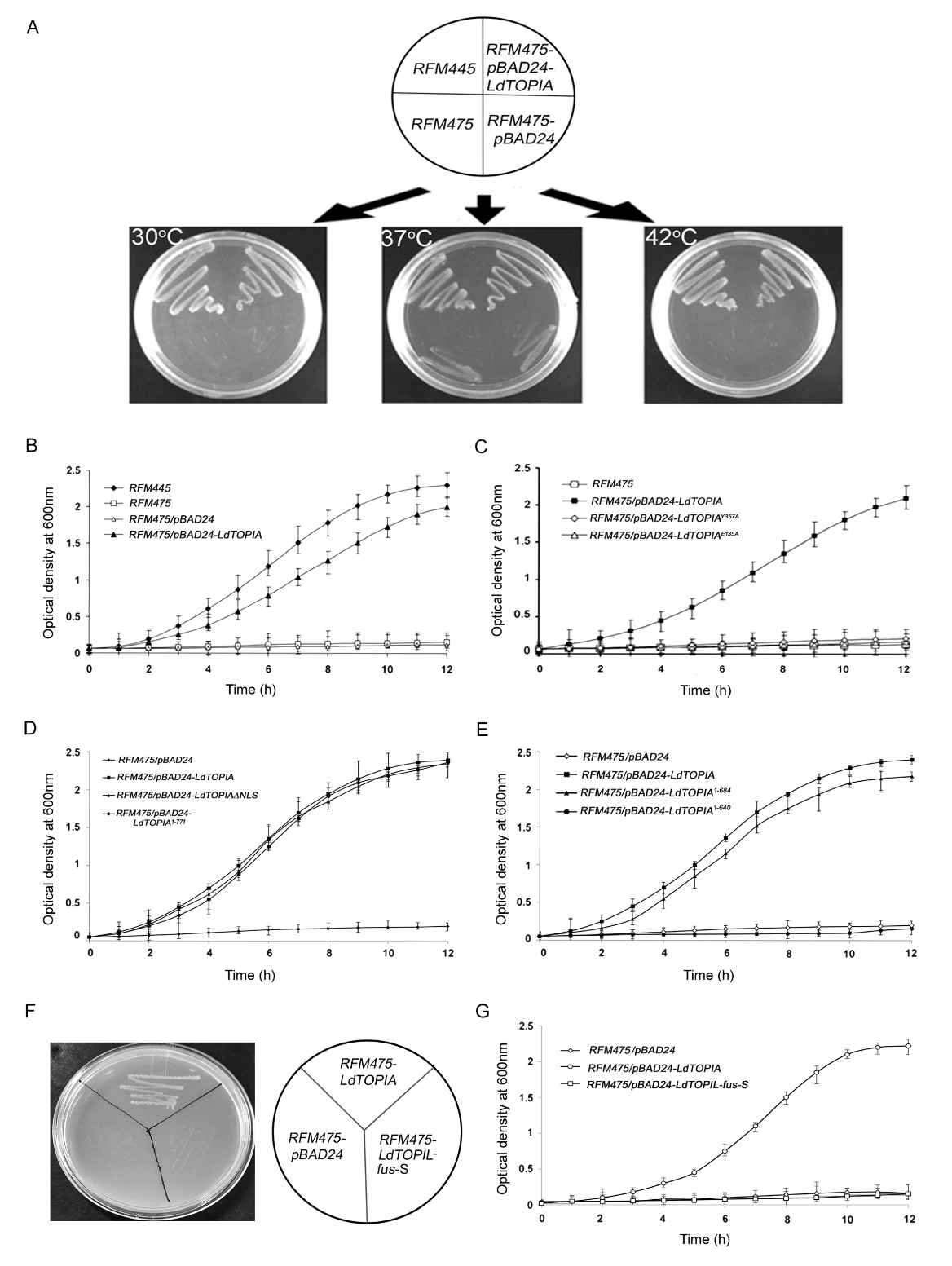


Figure S4: (A) To LB media agar plates containing ampicillin, the following strains RFM445 as positive control, RFM475 transformed with pBAD24, pBAD24-LdTOPIA and RFM475 were streaked and grown at permissible temperature 37°C and non-permissible 30°C and 42°C for 36hr. Growth kinetics in LB media for indicated RFM475 transformants. Graphical representation of growth kinetics of (B) RFM445, RFM475, RFM475/pBAD24 and RFM475/pBAD24-LdTOPIA. (n=3 and 3 biological replicates, mean ± SD). (C, D and E) Growth kinetics of complementation with SDM mutants RFM475-LdTOPIA^Y357A^, LdTOPIA^E135A^ and, deletion constructs LdTOPIA∆NLS, LdTOPIA1-771, LdTOPIA1-684 and LdTOPIA1-640 were done at 30°C. (n=3 and 3 biological replicates, mean ± SD) (F) RFM475 transformed with pBAD24-LdTOPIL-*fus-*S and grown at 30°C in LB agar plates. (G) Growth kinetics of the same. (n=3 and 3 biological replicates, mean ± SD)

**Figure S5**

**
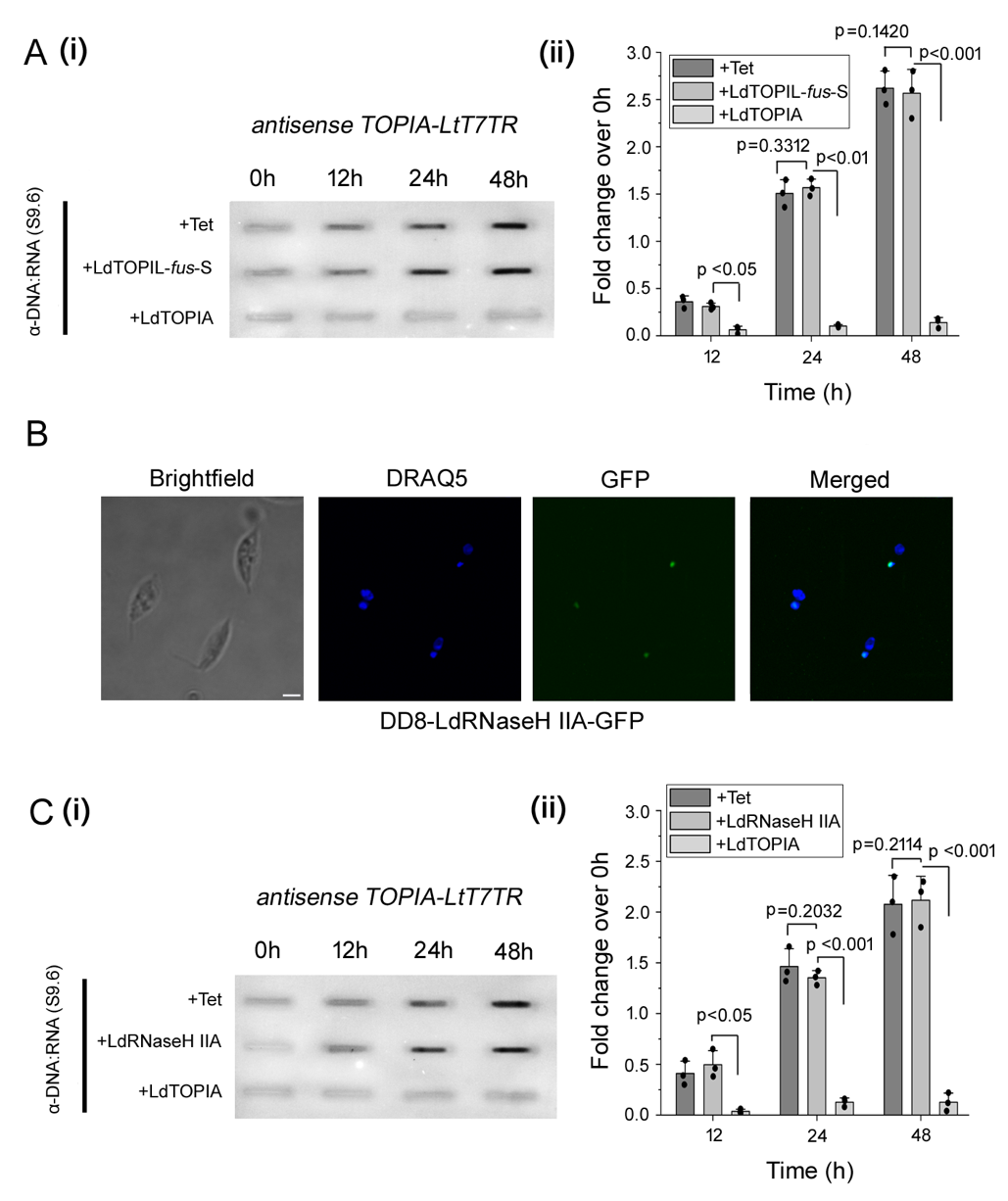
**

Figure S5: (A) (i) R-loops accumulation in complementation of antisense TOPIA-LtT7TR by LdTOPIL-*fus*-S were observed by Dot blot (ii) Densitometry of same samples. (n=3 and 2 biological replicate, mean ± SD) (B) Microscopic image of LdRNAseH IIA localization. Scale Bar, 5µm. LdRNaseH IIA – GFP fusion protein overexpressed inside DD8. Kinetoplast localization of LdRNAseH IIA observed by cyan color in merged image. (C) R-loops accumulation in complementation of antisense TOPIA-LtT7TR by LdRNaseH IIA were observed by Dot blot (ii) Densitometry of same samples. (n=3 and 2 biological replicates, mean ± SD)

**Figure S6**


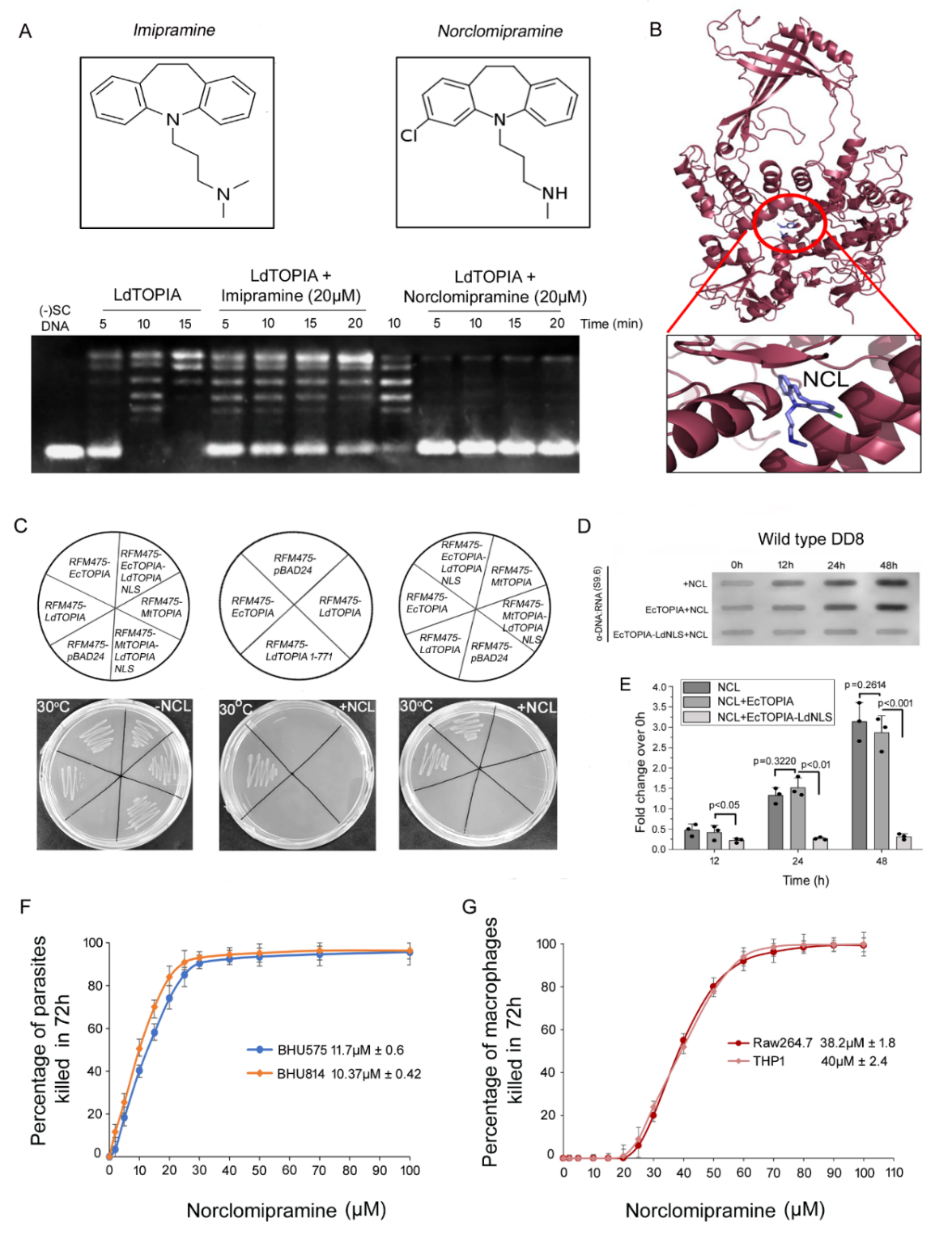


Figure S6: [A] (top panel) Structures of FDA approved tricyclic antidepressants Imipramine and Norclomipramine (NCL), [B] DNA relaxation assay carried out using LdTOPIA and (-) SC pBluescript DNA in absence and presence of imipramine and norclomipramine. (B) Molecular docking of norclomipramine onto the energy minimized homology modeled structure of LdTOPIA. ∆G= -33.12Kcal/mol. (C) Growth complementation in RFM475, in presence of 50µM NCL using transformed and arabinose induced LdTOPIA and EcTOPIA were done (middle panel). Complementation using RFM475 transformants expressing pBAD24 alone or LdTOPIA, LdTOPIA∆NLS, EcTOPIA, EcTOPIALdNLS, MtTOPIA and MtTOPIALdNLS in (left panel) absence and (right panel) presence of 50µM NCL were observed. (D) DRIB assay was carried out using genomic DNA isolated from EcTOPIA and EcTOPIA-LdNLS transfected DD8 parasites either untreated or NCL treated for indicated time points and (E) densitometric analysis of the same samples. (n=3 and 3 biological replicates, mean ± SD) (F) Anti-leishmanial activity of NCL on resistant clinical isolates BHU575 and BHU814 were observed by MTT assay. ((n=3 and 3 biological replicates, mean ± SD). (G) Cytotoxicity of NCL on macrophages (RAW264.7) and monocyte (THP1) were observed. (n=3 and 3 biological replicates, mean ± SD).
